# Spontaneous Water-Promoted Self-Aggregation of a Hydrophilic Gold(I) Complex Due to Ligand Sphere Rearrangement

**DOI:** 10.3390/molecules28155680

**Published:** 2023-07-27

**Authors:** Ainhoa Rodríguez-Gobernado, Daniel Blasco, Miguel Monge, José M. López-de-Luzuriaga

**Affiliations:** Departamento de Química, Centro de Investigación en Síntesis Química (CISQ), Universidad de La Rioja, Madre de Dios 53, 26006 Logroño, Spain; ainhoa.rodriguez@alum.unirioja.es

**Keywords:** gold(I), nucleobases, aurophilicity, aggregation-induced emission, nuclear magnetic resonance, photoluminescence, computational studies

## Abstract

Aggregating gold(I) complexes in solution through short aurophilic contacts promotes new photoluminescent deactivation pathways (aggregation-induced emission, AIE). The time dependence of spontaneous AIE is seldom studied. We examine the behavior of complex [Au(*N*^9^-hypoxanthinate)(PTA)] (**1**) in an aqueous solution with the aid of variable-temperature NMR, time-resolved UV–Vis and photoluminescence spectroscopy, and PGSE NMR. The studies suggest that partial ligand scrambling in favor of the ionic [Au(PTA)_2_][Au(*N*^9^-hypoxanthinate)_2_] pair followed by anion oligomerization takes place. The results are rationalized with the aid of computational calculations at the TD-DFT level of theory and IRI analysis of the electron density.

## 1. Introduction

The aqueous chemistry of linear gold(I) ([Xe] 5d^10^) is far less developed than that of its square-planar congener, gold(III) ([Xe] 5d^8^). The higher Pearson softness of gold(I) relative to gold(III) makes it less suitable for water coordination and thus disproportionate to give metallic gold(0) and gold(III). However, if the unstable gold(I) center is protected with suitable ligands, the complexes are stable in aqueous media and water-enriched mixtures [1]. This allows the exploitation of the interesting and characteristic properties of gold(I), e.g., catalytical [2,3,4,5,6,7,8] and biological [9,10,11] activity in these appealing media.

As an instance, gold(I) complexes have been popularized during the last years as Aggregation-Induced Emission Luminogens (AIEgens) [12] thanks to the ability of gold(I) to self-aggregate through short aurophilic contacts of <3.2 Å [13,14,15]. Gold(I) AIEgens essentially comprise highly hydrophobic complexes, e.g., pentafluorophenylgold(I) units bound to alkyl- or arylisocyanide ligands featuring long alkyl spacers or chains [16]. Aggregation is commonly forced by adding water to a neat solution of AIEgen. The spatial proximity of the gold(I) atoms in the nanosized aggregates gives rise to new excited states accessible by optical excitation, switching on new photoluminescent deactivation pathways. The degree of aggregation and the photoluminescence intensity are controlled by a balance between hydrophobicity and hydrophilicity.

There are fewer examples where AIE is achieved in gold(I) chemistry, not by modifying the solvent composition but spontaneously to gain thermodynamic stability. These include spontaneous self-assembly, as in the case of metallogelation [15,17,18], and ligand rearrangement conducive to diauracycles for releasing strain [19] or forming stabler species [20].

**Figure 1:**
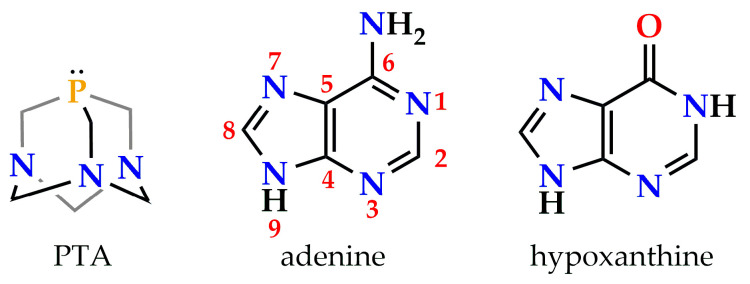
Molecular structures of PTA, adenine (canonical numbering in red) and hypoxanthine.

In this context, the water-soluble phosphine 1,3,5-triaza-7-phosphaadamantane (PTA; see Figure 1, left) [21] is widely employed as a ligand because of its small cone angle of 103°, the absence of π-delocalized electron density, and its extremely rich chemistry [22,23,24]. These properties allow gold(I)⋯gold(I) aggregation and the observation of AIE unencumbered by intraligand transitions. There are numerous studies on the self-aggregation of discrete gold(I) complexes of PTA and DAPTA (3,7-diacetyl-1,3,7-triaza-5-fosfabiciclo[3.3.1]nonane) [25,26] by Rodríguez’s group; see [27,28,29] as examples. However, the time dimension of the process is seldom studied. We reported in 2021 that complex [Au(*N*^9^-adeninate)(PTA)], an auration product of the natural purinic nucleobase adenine (see Figure 1, center), experiences a spontaneous supramolecular rearrangement in water solution leading to broad red phosphorescence centered at 700 nm [30]. Aurophilic dimers further stabilized by C-H⋯π interactions were proposed as a source of phosphorescence, in contrast to the initial blue-fluorescent hydrogen-bonded dimers. The rearrangement process occurs even within the semirigid fibrous matrix of a hydrometallogel.

We herein expand our previous study, considering the natural oxypurinic nucleobase hypoxanthine (6-hydroxypurine; see Figure 1, right). Hypoxanthine is an intermediate in adenine biosynthesis and the major product of the *C*^2^-deamination of guanine. The substitution of the *C*^6^-amino group of adenine by a *C*^6^-hydroxy one gives rise to a different balance and pattern of supramolecular hydrogen bond interactions. There are fewer examples of metal complexes of hypoxanthine, or oxypurines in general, than of adenine [31,32]. The spontaneous rearrangement process of complex [Au(*N*^9^-hypoxanthinate)(PTA)] (**1**) taking place exclusively in water solution is studied using time-resolved spectroscopical techniques, TD-DFT computational calculations, and IRI electron density topological analysis.

## 2. Results and Discussion

### 2.1. Synthesis and Characterization of Complex [Au(N^9^-Hypoxanthinate)(PTA)] (***1***)

**Figure 2:**
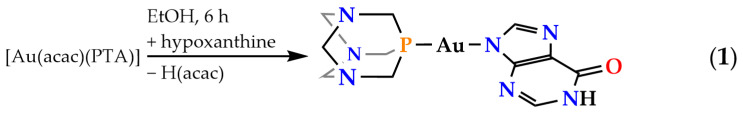
Synthesis of complex **1**.

Complex [Au(*N*^9^-hypoxanthinate)(PTA)] (**1**) was prepared in a single step by coordinating a [Au(PTA)]^+^ unit to the hypoxanthinate anion formed in situ by deprotonation of hypoxanthine with the acetylacetonate [(acac)^−^] ligand of [Au(acac)(PTA)] (see Figure 2). A *N*^9^ coordination mode of the [Au(PTA)]^+^ fragment to hypoxanthinate is proposed for complex **1** by analogy with the X-ray structure of other reported gold(I) complexes of the adeninate anion [18,30,33,34,35]. *N*^9^ is also the most basic position of purines. Complex **1** is freely soluble in water, where it experiences a spontaneous color change at room temperature associated with a nascent red photoluminescent emission (see below). It is also soluble in methanol, sparingly soluble in ethanol, and barely soluble in organic solvents such as dichloromethane, chloroform, diethylether, and *n*-hexane.

**Figure 3:**
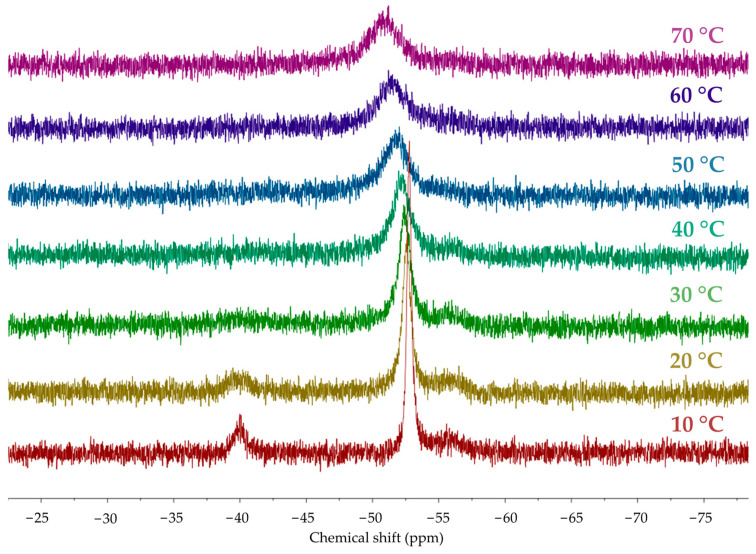
Collection of ^31^P{^1^H} NMR spectra of complex **1** (25 mM in D_2_O) at temperatures ranging from 10 °C to 70 °C.

**Figure 4:**
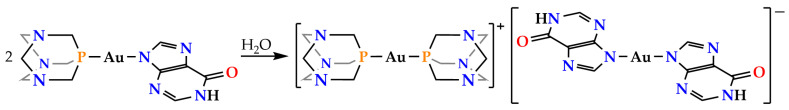
Proposed ion pair formation by ligand rearrangement in aqueous solution.

The presence of hypoxanthine as a ligand in complex **1** is suggested by the observed characteristic ν(C=O) (1682 cm^−1^) and ν(C=N) (1621 cm^−1^) stretching vibrations of the nucleobase and the loss of a broad ν(N-H) absorption centered at 2750 cm^−1^ in the UATR–FTIR spectrum (see Figures S1 and S2). The ^1^H NMR [ν_0_ of 400 MHz, in deuterium oxide (D_2_O); see Figure S3] nucleobase resonances are observed as broadened singlets at 7.99 (C^8^H) and 7.85 (C^2^H) ppm, deshielded with respect to free hypoxanthine [8.21 ppm (C^8^H), 8.19 ppm (C^2^H); see Figure S4]. Also, the ^1^H NMR phosphine signals appear as an AB system (lower rim, NCH_2_N) in the interval 4.46–4.56 ppm and as a virtual singlet (upper rim, PCH_2_N) centered at 4.30 ppm. Curiously, no effective ^2^J_PH_ coupling is detected, which seems to be characteristic of (PTA)gold(I) complexes [22]. The ^31^P{^1^H} NMR spectrum (ν_0_ of 162 MHz, in D_2_O; see Figure S5) is complicated with additional unexpected signals, but a major singlet at −52.63 ppm suggests one type of [Au(PTA)]^+^ coordination mode for the hypoxanthinate ligand. These spectra were recorded at temperatures ranging from 10 °C to 70 °C (see Figure 3 and Figure S6). The low freezing point of D_2_O restricted the temperature interval, precluding collection at lower temperatures. Up to three signals are observed in the ^31^P{^1^H} NMR spectrum at 10 °C. The downfield signal at −39.98 ppm is isochronous with that of the [Au(PTA)_2_]^+^ cation (−39.77 ppm, see below). In contrast, the upfield one at −55.56 ppm is assigned to coordination products of [Au(PTA)]^+^ to hypoxanthinate different from that of *N*^9^. The presence of [Au(PTA)_2_]^+^ can only be explained if the ligand scrambling process of Figure 4 is considered. In this process, two neutral molecules of complex **1** interchange their ligands. This renders the homoleptic ions [Au(PTA)_2_]^+^ and [Au(*N*^9^-hypoxanthinate)_2_]^−^. Indeed, the high polarity of water favors the partial formation of an ionic species. When the temperature is increased to 70 °C, the signals coalesce, averaging the chemical shifts to −51.21 ppm. This suggests that the different species are related by a thermal equilibrium.

The molecular peaks of [Au(*N*^9^-hypoxanthinate)(PTA)]^+^ (490 Da) and [Au(PTA)_2_]^+^ (511 Da) are observed in the MALDI-MS(+) spectrum (see Figures S7–S9). Other peaks of species of higher nuclearity are also observed, such as [Au_2_(hypoxanthinate)(PTA)_2_]^+^ (843 Da) and the sodium adduct {[Au_3_(hypoxanthinate)_2_(PTA)_2_]+Na}^+^ (1196 Da). These are produced by the stepwise addition of neutral [Au(hypoxanthinate)] units to [Au(PTA)_2_]^+^ under the harsh measurement conditions of MS. The aged solutions of complex **1** feature a peak of an even higher nuclearity, [Au_4_(hypoxanthinate)_3_(PTA)_2_]^+^ (1528 Da).

The freshly prepared water solutions of complex **1** behave as an expected non-electrolyte. However, its molar conductivity experiences a slight increase from 24.06 to 34.34 cm^2^ Ω^−1^ mol^−1^ during a 54-h period (see Figure S10).

These experimental findings, for example, the presence of [Au(PTA)_2_]^+^ in the ^31^P{^1^H} NMR spectrum of complex **1**, the detection of high-nuclearity species in the MALDI-MS(+) spectrum, the increase in the molar conductivity, and particularly the spontaneous color change of the solution (see below), reveal that complex **1** is not static in water solution.

### 2.2. Synthesis and Characterization of Complexes [Au(PTA)_2_](ClO_4_) (**2**) and (NBu_4_)[Au(N^9^-Hypoxanthinate)_2_] (***3***)

**Figure 5:**
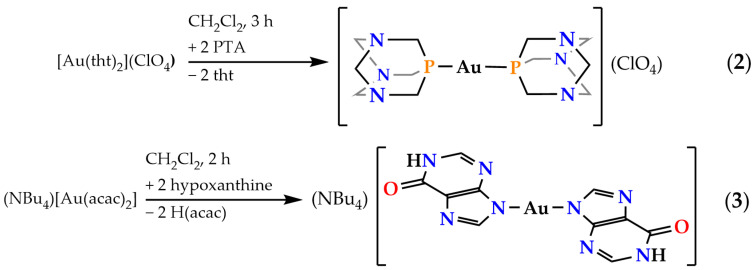
Syntheses of complexes **2** and **3**.

The homoleptic congeners of complex **1**, namely, [Au(PTA)_2_]^+^ cation and [Au(*N*^9^-hypoxanthinate)_2_]^−^ anion, as perchlorate (complex **2**) and tetrabutylammonium (complex **3**) salts, respectively, have also been prepared. Complex [Au(PTA)_2_](ClO_4_) (**2**) was prepared by displacement of the two labile tetrahydrothiophene (tht) ligands of [Au(tht)_2_](ClO_4_) with two equivalents of PTA in dichloromethane (see Figure 5, top). Complex (NBu_4_)[Au(*N*^9^-hypoxanthinate)_2_] (**3**) was prepared similarly to **1** by adding two equivalents of hypoxanthine to a solution of (NBu_4_)[Au(acac)_2_] in absolute ethanol (see Figure 5, bottom). Complex **2** is soluble in water, sparingly soluble in methanol and ethanol, and insoluble in organic solvents such as acetone, diethylether, and *n*-hexane. Complex **3** is sparingly soluble in water and insoluble in organic solvents such as dichloromethane, diethylether, and *n*-hexane. Interestingly, neither complex **2** nor complex **3** experience color changes or photoluminescence appearances in water solutions.

The spectroscopic measurements are consistent with the proposed stoichiometries for both complexes (see Supplementary Materials). Particularly for complex **2**, a sharp singlet at −39.77 ppm in the ^31^P {^1^H} NMR spectrum (ν_0_ of 162 MHz, in D_2_O; see Figure S12) suggests the presence of equivalent phosphine ligands. The molecular peaks of both ions are observed in their respective MALDI-MS spectra: {511 Da for [Au(PTA)_2_]^+^ and 98 Da for (ClO_4_)^−^} (see Figures S13 and S14). The presence of bound hypoxanthinate ligands in complex **3** is suggested by the ν(C=O) (1663 cm^−1^) and (C=N) (1610 cm^−1^) stretching vibrations of the nucleobase in the UATR–FTIR spectrum of **3** (see Figure S16).

**Figure 6:**
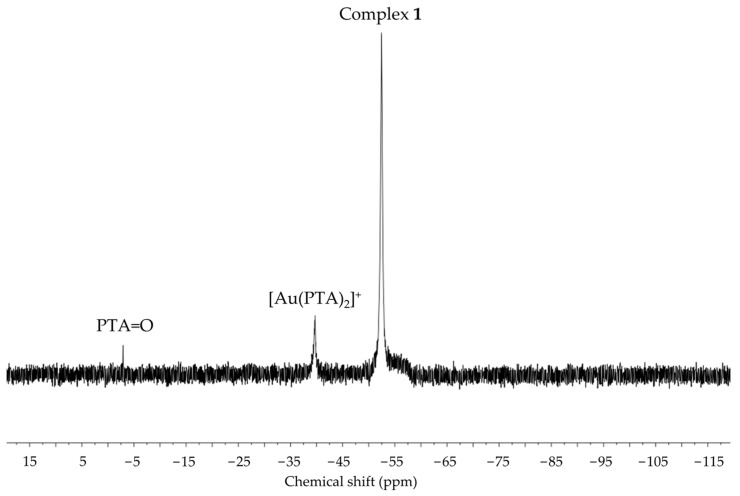
^31^P{^1^H} NMR spectra of the equimolecular mixture of complexes **2** and **3** in D_2_O.

To confirm whether the proposed equilibrium depicted in Figure 4 is indeed an equilibrium and if it can also be reached from the right side of the equation, an equimolecular mixture of complexes **2** and **3** in D_2_O has been characterized by variable-temperature ^1^H and ^31^P{^1^H} NMR (see Figure S20 and Figure 6, respectively). It can be observed that the ^31^P{^1^H} NMR spectrum of the mixture is fully different from that of neat complex **2** and is similar to that of **1**, featuring two major signals at −39.70 and −52.46 ppm, which correspond to [Au(PTA)_2_]^+^ and [Au(*N*^9^-hypoxanthinate)(PTA)], respectively. This confirms that the in situ formed ionic [Au(PTA)_2_][Au(^9^*N*-hypoxanthinate)_2_] pair equilibrates with neutral [Au(*N*^9^-hypoxanthinate)(PTA)] in water solution by ligand scrambling.

### 2.3. Photophysical Studies

**Figure 7:**
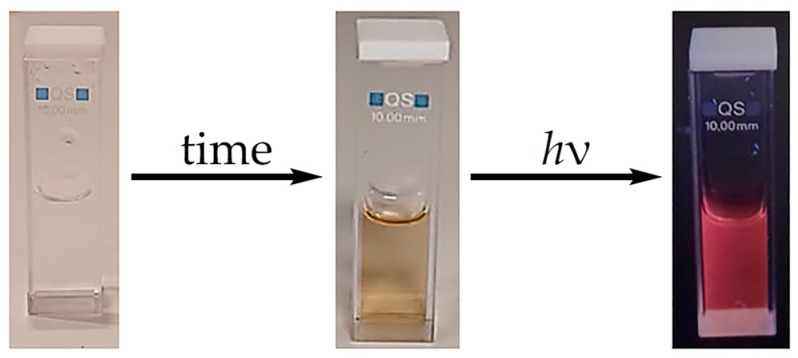
The color change of complex **1** in water solution (2 mM) upon aging.

As was advanced, the initially colorless water solutions of complex **1** turn to an amber color and emit red light (λ_ex_ of 365 nm) as a spontaneous result of aging (see Figure 7).

**Figure 8:**
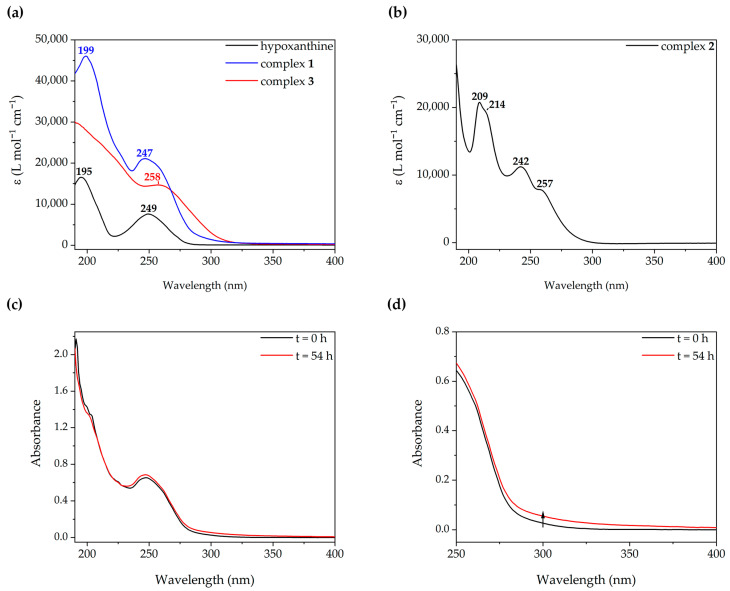
(**a**) Superimposition of the absorption spectra of hypoxanthine (black) and complexes **1** (blue) and **3** (red). (**b**) Absorption spectrum of complex **2**. (**c**) Absorption spectra of complex **1** at 0 (black) and 54 h (red). (**d**) Ampliation of the low energy region of (**c**). The spectra were measured in a water solution at a concentration of 50 μM.

The UV–Vis spectra of freshly prepared solutions of complexes **1**–**3** (50 μM in water solution) are plotted in Figure 8a,b. The spectrum of hypoxanthine is also included for comparative purposes. The spectrum of complex **1** features two distinct asymmetric absorptions at 245 and 200 nm. The energy of the bands is coincident with those of free hypoxanthine, suggesting an intraligand origin for them, with a slight perturbation arising from the [Au(PTA)]^+^ fragment. This is confirmed by computational calculations at the TD-DFT level of theory (see below). The coordination of two hypoxanthinate ligands to a single gold(I) center has a more significant impact on the spectrum. The spectral profile of complex **3** still features two absorptions, but these are broader and less defined. Notably, the band edge is redshifted. The spectrum of complex **2** shows two structured absorptions at 242 and 209 nm, with shoulders at 257 and 214 nm. The aqueous solutions of PTA do not show absorptions at wavelengths longer than 210 nm [36], so a metal-perturbed intraligand origin is proposed.

The amber color of the aged solutions of complex **1** relates to the subtle band edge redshift and increment of absorbance measured after 48 h (see Figure 8c,d). Its origin may be the steady formation of the [Au(*N*^9^-hypoxanthinate)_2_]^−^ anion since the band edge of **3** is red-shifted with respect to that of **1**. The 48-h spectrum of **1** is intermediate between 0-h **1** and **3**.

**Figure 9:**
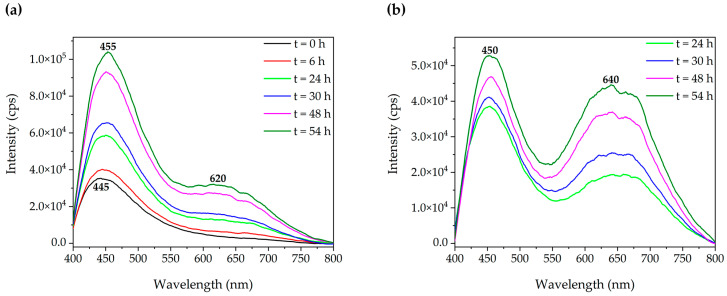
Emission spectrum of complex **1** (2 mM in aqueous solution) at different times with (**a**) an excitation wavelength of 320 nm and (**b**) an excitation wavelength of 340 nm.

The photoemission spectrum of complex **1** (2 mM in water solution) has been recorded at different times for 54 h (see Figure 9). Initially, the emission spectrum (λ_ex_ of 320 nm) featured a broad asymmetric band with a maximum of 445 nm and an emission lifetime of 73 ns. As time passed, a new lower-energy band (620 nm) with a longer lifetime of 628 ns as a shoulder of the former appeared. The excitation spectra of both bands overlap (see Figure S21); however, the low-energy band can be better resolved if the excitation wavelength is moved to 340 nm (see Figure 9b). During the 54-h period, an intensity increase in both bands is observed.

This time-dependent variation of the photoemission in aqueous solution is ascribed to the formation of fixed-size small aurophilic oligomers (see below), which may be formed from discrete neutral molecules of complex **1** or the ions originated from the partial ligand rearrangement proposed in Figure 4.

### 2.4. Pulsed-Field Gradient Spin Echo NMR Studies

The translational diffusion coefficients (*D_t_*) of the ligands conforming to complex **1** were measured by pulsed-field gradient spin-echo (PGSE) ^1^H NMR (ν_0_ of 400 MHz, 25 mM in D_2_O) [37,38]. The hydrodynamic radius (*r*_H_) and the molecular size of the diffusing species are inversely proportional to *D_t,_* as expressed by the Stokes–Einstein equation. The *D_t_* and derived *r*_H_ of characteristic ^1^H NMR signals of the PTA and hypoxanthinate ligands measured from a freshly prepared sample and after 48 h are compared in Table 1.

Table 1 shows that both ligands have different *D_t_* irrespective of the sample preparation time, suggesting they do not belong to the same molecule. Instead, the ligands may be bound to different gold(I) atoms, diffusing differently. This fact agrees with the formation of the ionic pair due to the redistribution of ligands proposed to occur in an aqueous solution. Although the time variations of *D_t_* and *r*_H_ are small in both cases, the ones of PTA are almost negligible. Thus, the PTA-containing species, namely [Au(*N*^9^-hypoxanthinate)(PTA)] (**1**) and [Au(PTA)_2_]^+^, do not seem to participate in the oligomerization processes.

### 2.5. Computational Studies

**Figure 10:**
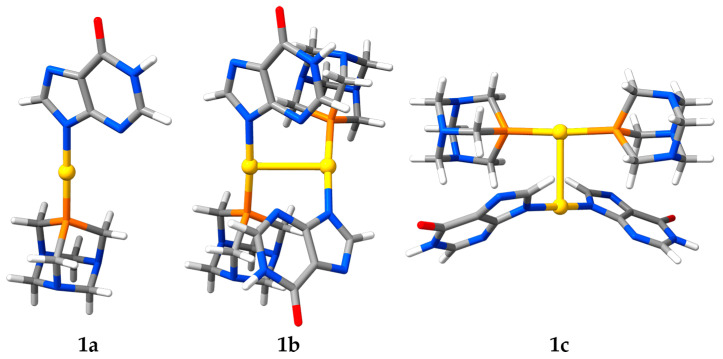
Optimized structures of models **1a**–**1c**. Color code: C, grey; H, white; Au, yellow; N, blue; O, red; P, orange.

**Figure 11:**
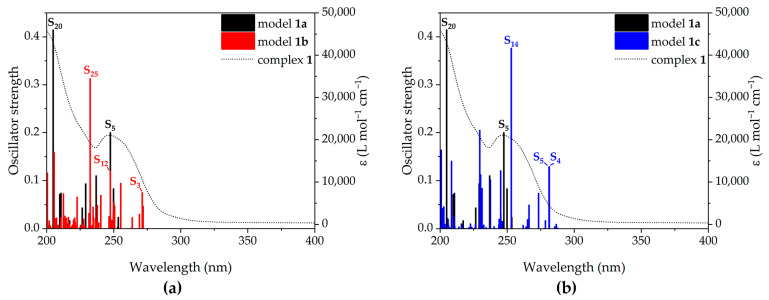
Overlay of the UV–Vis spectrum of complex **1** in water (dotted line) with the calculated vertical singlet excitations of (**a**) models **1a** (black bars) and **1b** (red bars), and (**b**) models **1a** (black bars) and **1c** (blue bars).

The time-dependent optical properties of **1** in aqueous solution are explained with the aid of three computational models. On the one hand, there is the [Au(*N*^9^-hypoxanthinate)(PTA)] neutral monomer, model **1a**. On the other hand, there are the [Au(*N*^9^-hypoxanthinate)(PTA)]_2_ neutral and [Au(PTA)_2_][Au(*N*^9^-hypoxanthinate)_2_] ionic dimers, models **1b** and **1c**, respectively (see Figure 10). An aurophilically bound dimer for **1b** instead of a hydrogen-bonded one was chosen to allow a more reasonable comparison with **1c**. The structures of models **1a**–**1c** were optimized at the RI-DFT/PBE0-D3(BJ)/def2-TZVP level of theory with a continuum solvation model representing water. A TD-DFT calculation of the first singlet and triplet vertical excitations was carried out. The energies, oscillator strengths, and orbital contributions of a selection of these excitations are collected in Table 2. An overlay of the experimental UV–Vis spectrum of complex **1** in water with the calculated singlet excitations of models **1a**–**1c** is plotted in Figure 11. The molecular orbitals contributing to the excitations are collected in the Supplementary Materials.

The TD-DFT calculation on model **1a** predicts the two most intense transitions at 247 and 205 nm, corresponding to the two absorption bands observed in the UV–Vis spectrum of complex **1**. Thus, the low-energy band is assigned to a mixture of HOMO to LUMO and HOMO-1 to LUMO+1 transitions consisting of charge transfers from the PTA ligand to the gold(I) center. In contrast, the high-energy band is assigned to a HOMO-4 to LUMO metal-perturbed intraligand transition within the hypoxanthine ligand, with a second arising from a HOMO-1 to LUMO+4 ligand-to-metal charge transfer.

The TD-DFT predictions for models **1b** and **1c** are more complicated, showing increased transitions covering the whole UV–Vis spectrum of complex **1**. It is noteworthy that the transitions to the first excited singlet state are less energetic for the dimerized models (272 nm, **1b**; 288 nm, **1c**) than for the monomer one (253 nm). This explains the increased absorbance of the low-energy tail of the UV–Vis spectrum of **1** upon oligomerization. The most intense transition among the lower-energy ones of model **1b** consists of a mixture of up to three charge transfers from the PTA-centered HOMO-1, HOMO-2, and HOMO to LUMO, which has a major contribution on the intermetallic axis. The analogous transitions for model **1c**, which appear almost degenerate due to the *C*_2_ symmetry of the model, feature a similar origin to that of **1b**. However, in this case, the gold(I) atom bound to the PTA ligands has more weight in the transition.

**Figure 12:**
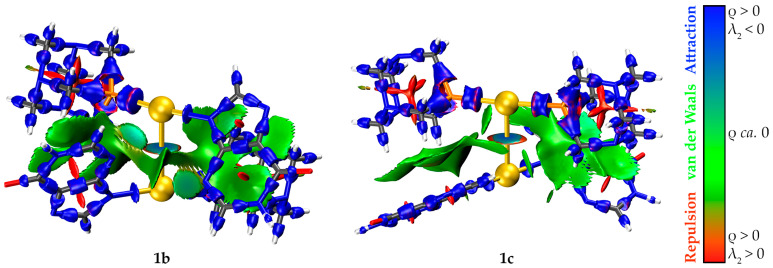
IRI isosurfaces (isovalue of 1.1) of models **1b** and **1c**. Color code: C, grey; H, white; Au, yellow; N, blue; O, red; P, orange.

Finally, the optimized electron density was studied using the interaction region indicator (IRI). The RGB-scale mapping of the IRI isosurfaces with the electron density (ρ) weighted by the sign of the second largest eigenvalue of the Hessian (λ_2_) is a powerful tool to simultaneously reveal and visualize both covalent and non-covalent interactions at a cheap computational cost. The IRI isosurfaces (isovalue of 1.1) of models **1b** and **1c** are plotted in Figure 12. The sign of λ_2_ distinguishes between attractive (blue), van der Waals (green), and repulsive (red) interactions.

A strong aurophilic interaction as a green-to-blue disk between the gold(I) atoms and several C-H⋯π interactions as a green surface between the C-H bonds of PTA and the π-electron density of the hypoxanthinate ligands keep the fragments bound in both cases.

## 3. Materials and Methods

### 3.1. General Procedures

All reactions were carried out at room temperature in an open-air atmosphere. Complexes [Au(acac)(PTA)] [35] and [Au(tht)_2_](ClO_4_) [39] were prepared as described in the bibliography, whereas (NBu_4_)[Au(acac)_2_] was prepared as described in [40] but employing (NBu_4_)[AuCl_2_] instead of [N(PPh_3_)_2_][AuCl_2_]. Hypoxanthine and 1,3,5-triazaphosphaadamantane were purchased from Sigma-Aldrich (Madrid, Spain) and employed as received. The Milli-Q water employed in photoluminescence measurements was saturated with nitrogen gas by continuous bubbling for 10 min.

### 3.2. Instrumentation

FTIR spectra were measured with a Perkin-Elmer Two (Perkin-Elmer, Waltham, MA, USA) spectrophotometer equipped with a diamond crystal UATR accessory. ^1^H, ^31^P{^1^H}, and ^1^H PGSE NMR spectra were measured with a Bruker AVANCE 400 (Bruker Corporation, Fällanden, Switzerland) spectrometer in D_2_O solution. Chemical shifts are quoted relative to SiMe_4_ (^1^H, external) and H_3_PO_4_ 85% in D_2_O (^31^P, external). ^1^H PGSE-NMR measurements were carried out with the doubly stimulated echo-pulsed sequence (Double STE) on a Bruker AVANCE 400 equipped with a BBI H-BB Z-GRD probe at 298 K without spinning at different times in D_2_O. Steady-state luminescence spectra were measured with a spectrofluorimeter of HORIBA Jobin Yvon Fluorolog-3 (HORIBA Jobin Yvon, Stow, MA, USA). Emission lifetimes with the time-correlated single photon counting technique were measured with the Datastation HUB (HORIBA Jobin Yvon) and a nanoLED (HORIBA Jobin Yvon) of 320 nm. Conductivities were measured in ca. 0.5 mM water solutions with a Jenway 4010 conductimeter (Jenway, Felsted, UK). MALDI-MS spectra in negative and positive modes were measured with a Bruker MicroTOF-Q spectrometer equipped with a MALDI-TOF ionization source (Bruker Corporation, Bremen, Germany). UV–Vis spectra in aqueous solution were measured with a LAMBDA 265 UV/Vis spectrophotometer (Perkin-Elmer).

### 3.3. Computational Details

The calculations were carried out with TURBOMOLE version 7.5 [41]. Models [Au(*N*^9^-hypoxanthinate)(PTA)] (**1a**), [Au(*N*^9^-hypoxanthinate)(PTA)]_2_ (**1b**), and [Au(PTA)_2_][Au(*N*^9^-hypoxanthinate)_2_] (**1c**) were built from scratch. The structures were preoptimized at the density functional theory (DFT) level [42] with the PBE0 functional [43,44,45], def2-SVP basis sets on all atoms [46], the 60 core electron def2-ECP [47] for gold, the resolution-of-the-identity (RI) approximation [48,49,50] to accelerate the calculations and the semiempirical D3(BJ) correction [51,52] to dispersion interactions. It was followed by a second optimization at the same level of theory but with the larger def2-TZVP basis sets [46] on all atoms. A final optimization with the conductor-like screening model (COSMO) approximation [53] of water (ε = 80.1) at the same level of theory was carried out. The first eighty singlets and the first triplet vertical excitations were calculated at the time-dependent DFT (TD-DFT) level [54,55,56,57] with the PBE0 functional [43,44,45], def2-TZVP basis sets [46] on all atoms, def2-ECP [47] for gold, and the COSMO approximation [53]. The optimized models and molecular orbitals were visualized and rendered using UCSF ChimeraX version 1.5 [58]. The interaction region indicator (IRI) [59] was computed with Multiwfn version 3.8 [60]. The IRI isosurfaces were plotted with VMD version 1.9.4a53 [61].

### 3.4. Synthesis of Complex [Au(N^9^-Hypoxanthinate)(PTA)] (***1***)

Hypoxanthine (white solid; 0.0310 g, 0.223 mmol) is added to a solution of [Au(acac)(PTA)] (beige solid; 0.1015 g, 0.224 mmol) in 20 mL of absolute ethanol. The suspension is stirred for 6 h. The mixture is rotary evaporated to a volume of 2 mL. [Au(*N*^9^-hypoxanthinate)(PTA)] (white solid; 0.0870 g, 0.178 mmol, 80%) precipitates by addition of 20 mL of *n*-hexane and is isolated by suction filtration and washed with three fractions of 5 mL of *n*-hexane.

ATR-FTIR, cm^−1^: ν(C=O) = 1682 (m), ν(C=N) = 1621 (s). ^1^H NMR (400 MHz, D_2_O, 298 K), ppm: 7.99 (s, 1H, C^8^H), 7.85 (s, 1H, C^2^H), 4.56–4.46 (AB q, 6H, NCH_2_N), 4.30 (ps, 6H, PCH_2_N). ^31^P{^1^H} NMR (162 MHz, D_2_O, 298 K), ppm: −52.63 (s, PTA). MALDI-MS(+), *m/z:* 490.1 [**1**+H]^+^ (calculated 490.1 Da), 511.2 [Au(PTA)_2_]^+^ (calculated 511.1 Da), 843.3 [Au_2_(*N*^9^-hypoxanthinate)(PTA)_2_]^+^ (calculated 843.1 Da).

### 3.5. Synthesis of Complex [Au(PTA)_2_](ClO_4_) (***2***)

PTA (white solid; 0.0803 g, 0.510 mmol) is added to a solution of [Au(tht)_2_](ClO_4_) (white solid; 0.1207 g, 0.255 mmol) in 15 mL of dichloromethane. The suspension is stirred for 3 h. The mixture is rotary evaporated to a volume of 2 mL. Complex [Au(PTA)_2_](ClO_4_) (white solid; 0.1450 g, 0.237 mmol, 93%) precipitates by addition of 15 mL of n-hexane and is isolated by suction filtration and washed with three fractions of 5 mL of n-hexane.

ATR-FTIR, cm^−1^: ν (C-H) = 2932 (s). ^1^H NMR (400 MHz, D_2_O, 298 K), ppm: 4.67–4.50 (AB q, 6H, NCH_2_N), 4.36 (ps, 6H, PCH_2_N). ^31^P{^1^H} NMR (162 MHz, D_2_O, 298 K), ppm: −39.77 (s, PTA). MALDI-MS(+), *m/z*: 511.2 [Au(PTA)_2_]^+^ (calculated 511.1 Da). MALDI-MS (-), *m/z*: 98.9 (ClO_4_^−^) (calculated 98.9 Da).

### 3.6. Synthesis of Complex (NBu_4_)[Au(N^9^-Hypoxanthinate)_2_] (***3***)

Hypoxanthine (white solid; 0.0229 g, 0.168 mmol) is added to a solution of (NBu_4_)[Au(acac)_2_] (white solid; 0.0534 g, 0.084 mmol) in 10 mL of absolute ethanol. The suspension is stirred for 2 h. The mixture is rotary evaporated to a volume of 2 mL. Complex (NBu_4_)[Au(*N*^9^-hypoxanthinate)_2_] (white solid, 0.0339 g, 0.048 mmol) precipitates by addition of 10 mL of diethyl ether and is isolated by suction filtration and washed with three fractions of 5 mL of diethyl ether.

ATR-FTIR, cm^−1^: ν(C=O) = 1663 (m), ν(C=N) = 1610 (s). ^1^H NMR (400 MHz, D_2_O, 298 K), ppm: 8.16 (s, 2H, C^8^H), 8.15 (s, 2H, C^2^H), 3.31 (ps, 8H, C**H**_2_CH_2_CH_2_CH_3_), 1.56 (ps, 8H, CH_2_C**H**_2_CH_2_CH_3_), 1.30 (ps, 8H, CH_2_CH_2_C**H**_2_CH_3_), 0.89 (ps, 12H, CH_2_CH_2_CH_2_C**H**_3_) MALDI-MS(+), *m/z:* 242.2 (NBu_4_^+^) (calculated 242.3 Da).

## 4. Conclusions

**Figure 13:**
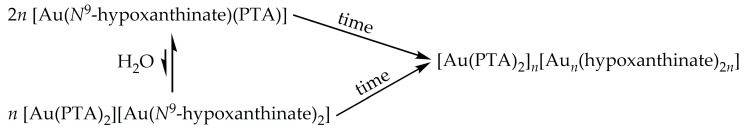
Proposed water-driven ligand rearrangement and time-dependent oligomerization scheme for complex **1** in water solution.

The efficient dipole solvation properties of water prompt the partial redistribution of the asymmetrically coordinated [Au(*N*^9^-hypoxanthinate)(PTA)] into the stable charged species [Au(*N*^9^-hypoxanthinate)_2_]^−^ and [Au(PTA)_2_]^+^. Afterwards, [Au(*N*^9^-hypoxanthinate)_2_]^−^ experiences an aggregation process as reflected by its decreasing *D_t_* upon aging. [Au(PTA)_2_]^+^ remains undisturbed in solution, equilibrating the negative electric charge of the supposed [Au_n_(hypoxanthinate)_2n_]^n−^ aggregate and stabilizing it by C-H⋯π interactions. The latter species is proposed to be the source of phosphorescence. However, an unequivocal assignment of the optical properties cannot be provided without conclusive structural data for complex **1** and the emissive products. In contrast to regular AIE, no poor solvent addition is needed to force gold(I)⋯gold(I) clustering and achieve photoemission. A tentative scheme of the processes occurring after dissolving complex **1** in water is given in Figure 13.

## Figures and Tables

**Table 1:** Translational diffusion coefficients (in m s^−1^) and derived hydrodynamic radii (in Å) of complex **1** at 0 and 48 h.

Time (h)	PTA Signal (4.32 ppm)		Hypoxanthinate Signal (8.01 ppm)	
	*D_t_*·10^−10^ (m s^−1^)	*r*_H_ (Å)	*D_t_*·10^−10^ (m s^−1^)	*r*_H_ (Å)
0	4.16	4.93	5.80	3.54
48	4.13	4.96	5.51	3.72

**Table 2:** Energies (in nm), oscillator strengths (dimensionless), and orbital contributions (in %) of selected vertical singlet and triplet excitations of models **1a**–**1c**.

Excitation	Energy (nm)	Oscillator Strength	Orbital Contributions (%)			
Model **1a**						
S_1_ ← S_0_	253	0.00	HOMO-1 (85)	→	LUMO (87)	(51.8)
			HOMO (86)	→	LUMO+1 (88)	(32.7)
S_5_ ← S_0_	247	0.20	HOMO-1 (85)	→	LUMO+1 (88)	(68.6)
			HOMO (86)	→	LUMO (87)	(20.5)
S_20_ ← S_0_	205	0.41	HOMO-4 (82)	→	LUMO (87)	(74.7)
			HOMO-1 (85)	→	LUMO+4 (91)	(13.2)
T_1_ ← S_0_	336	- ^a^	HOMO-2 (84)	→	LUMO+2 (89)	(57.2)
			HOMO-2 (84)	→	LUMO (87)	(34.5)
Model **1b**						
S_1_ ← S_0_	272	0.05	HOMO (172)	→	LUMO (173)	(77.7)
			HOMO-1 (171)	→	LUMO (173)	(18.1)
S_3_ ← S_0_	271	0.08	HOMO-1 (171)	→	LUMO (173)	(63.8)
			HOMO-2 (170)	→	LUMO (173)	(15.1)
			HOMO (172)	→	LUMO (173)	(14.7)
S_12_ ← S_0_	247	0.12	HOMO (172)	→	LUMO+1 (174)	(46.5)
			HOMO-4 (168)	→	LUMO+1 (174)	(23.6)
S_25_ ← S_0_	232	0.31	HOMO-5 (167)	→	LUMO+3 (176)	(43.4)
			HOMO-4 (168)	→	LUMO+4 (177)	(34.1)
T_1_ ← S_0_	337	- ^a^	HOMO-4 (168)	→	LUMO+1 (174)	(32.9)
			HOMO-5 (167)	→	LUMO+2 (175)	(31.8)
Model **1c**						
S_1_ ← S_0_	288	0.00	HOMO (172)	→	LUMO+1 (174)	(96.1)
S_4_ ← S_0_	281	0.13	HOMO-2 (170)	→	LUMO+1 (174)	(60.4)
			HOMO (172)	→	LUMO (173)	(19.8)
			HOMO-1 (171)	→	LUMO+1 (174)	(16.8)
S_5_ ← S_0_	281	0.13	HOMO (172)	→	LUMO (173)	(44.4)
			HOMO-2 (170)	→	LUMO+1 (174)	(34.9)
			HOMO-1 (171)	→	LUMO+1 (174)	(17.5)
S_14_ ← S_0_	253	0.36	HOMO-6 (166)	→	LUMO (173)	(90.5)
T_1_ ← S_0_	336	- ^a^	HOMO-4 (168)	→	LUMO+2 (175)	(42.5)
			HOMO-5 (167)	→	LUMO+3 (176)	(40.8)

^a^ We cannot presently estimate the strength given by spin–orbit effects to the triplet transitions.

## Data Availability

Not applicable.

## Supplementary Materials

The following supporting information can be downloaded at: https://www.mdpi.com/article/10.3390/molecules28155680/s1, Figure S1: UATR–FTIR spectrum of complex **1**. Figure S2: UATR–FTIR spectrum of hypoxanthine. Figure S3: ^1^H NMR (400 MHz, D_2_O) spectrum of complex **1**. Figure S4: ^1^H NMR (400 MHz, D_2_O) spectrum of hypoxanthine. Figure S5: ^31^P{^1^H} NMR (162 MHz, D_2_O) spectrum of complex **1**. Figure S6: Collection of ^1^H NMR spectra of complex **1** in D_2_O (25 mM) at different temperatures. Figure S7: MALDI-MS(+) spectrum of complex **1**. Figure S8: MALDI-MS(+) spectrum of an aged solution of complex **1**. Figure S9: MALDI-MS(-) spectrum of complex **1**. Figure S10: Molar conductivity in aqueous solution of complex **1** at different times. Figure S11: ^1^H NMR (400 MHz, D_2_O) spectrum of complex **2**. Figure S12: ^31^P{^1^H} NMR (162 MHz, D_2_O) spectrum of complex **2**. Figure S13: MALDI-MS(+) spectrum of complex **2**. Figure S14: MALDI-MS(-) spectrum of complex **2**. Figure S15: UATR–FTIR spectrum of complex **2**. Figure S16: UATR–FTIR spectrum of complex **3**. Figure S17: MALDI-MS(+) spectrum of complex **3**. Figure S18: MALDI-MS(-) spectrum of complex **3**. Figure S19: ^1^H NMR (400 MHz, D_2_O) spectrum of complex **3**. Figure S20: ^1^H NMR (400 MHz, D_2_O) spectrum of the equimolecular mixture of complexes **2** and **3**. Figure S21: Excitation spectra of complex **1** in aqueous solution with an emission wavelength of 450 nm (black line) or 650 nm (blue line). Figure S22: Molecular orbitals involved in the S_1_ transition of model **1a**. Figure S23: Molecular orbitals involved in the S_5_ transition of model **1a**. Figure S24: Molecular orbitals involved in the S_20_ transition of model **1a**. Figure S25: Molecular orbitals involved in the T_1_ transition of model **1a**. Figure S26: Molecular orbitals involved in the S_1_ transition of model **1b**. Figure S27: Molecular orbitals involved in the S_3_ transition of model **1b**. Figure S28: Molecular orbitals involved in the S_12_ transition of model **1b**. Figure S29: Molecular orbitals involved in the S_25_ transition of model **1b**. Figure S30: Molecular orbitals involved in the T_1_ transition of model **1b**. Figure S31: Molecular orbitals involved in the S_1_ transition of model **1c**. Figure S32: Molecular orbitals involved in the S_4_ transition of model **1c**. Figure S33: Molecular orbitals involved in the S_5_ transition of model **1c**. Figure S34: Molecular orbitals involved in the S_14_ transition of model **1c**. Figure S35: Molecular orbitals involved in the T_1_ transition of model **1c**. Table S1. Cartesian coordinates of the optimized structure [RI-DFT/PBE0-D3(BJ)/COSMO(ε = 80.1)/def2-TZVP/def2-ECP(Au)] of model **1a**. Table S2. Cartesian coordinates of the optimized structure [RI-DFT/PBE0-D3(BJ)/COSMO(ε = 80.1)/def2-TZVP/def2-ECP(Au)] of model **1b**. Table S3. Cartesian coordinates of the optimized structure [RI-DFT/PBE0-D3(BJ)/COSMO(ε = 80.1)/def2-TZVP/def2-ECP(Au)] of model **1c**.

## Sample Availability

Samples of the compounds are available from the authors.
