# Supplementary material for: Spontaneous Water-Promoted Self-Aggregation of a Hydrophilic Gold(I) Complex Due to Ligand Sphere Rearrangement
Source: Molecules. 2023 Jul 27;28(15):5680. doi: 10.3390/molecules28155680 (PMC10420115; doi:10.3390/molecules28155680)
Supplement: Supplementary file 1 [file molecules-28-05680-s001.zip › molecules-2519246-supplementary.pdf]

molecules

MDPI

Supplementary Materials

Spontaneous Water-Promoted Self-Aggregation of a Hydrophilic Gold(I) Complex Due to Ligand Sphere Rearrangement

Ainhoa Rodríguez-Gobernado, Daniel Blasco, * Miguel Monge * and José M. López-de-Luzuriaga *

Departamento de Química, Centro de Investigación en Síntesis Química (CISQ), Universidad de La Rioja, Madre de Dios 53, 26006 Logroño, Spain; ainhoa.rodriguez@alum.unirioja.es

* Correspondence: daniel.blascos@unirioja.es (D.B.); miguel.monge@unirioja.es (M.M.); josemaria.lopez@unirioja.es (J.M.L.-d.-L.)

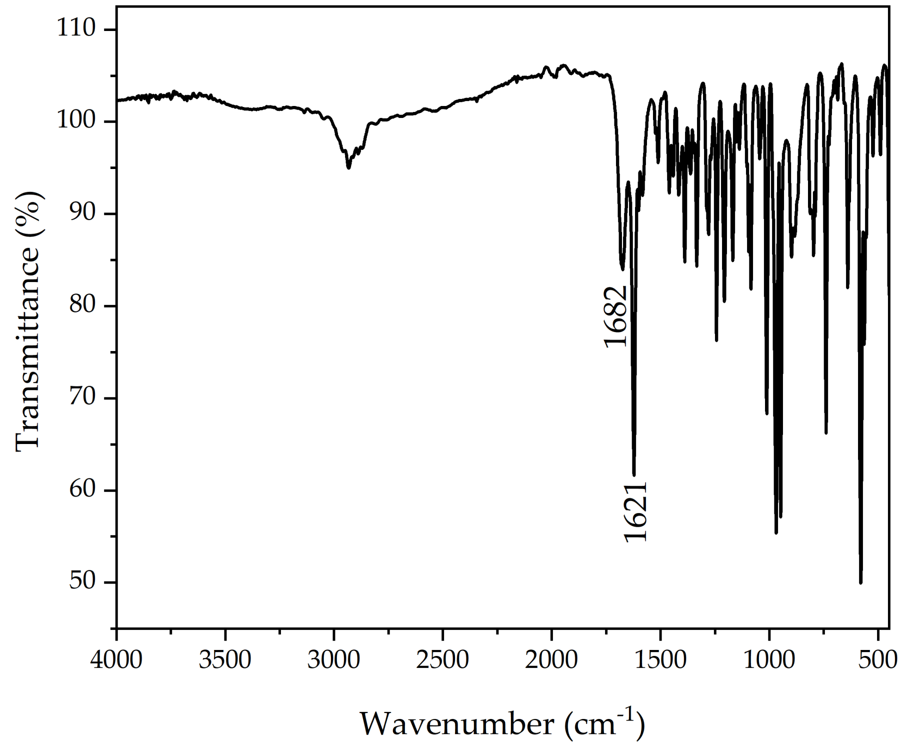

Figure S1. UATR-FTIR spectrum of complex **1**.

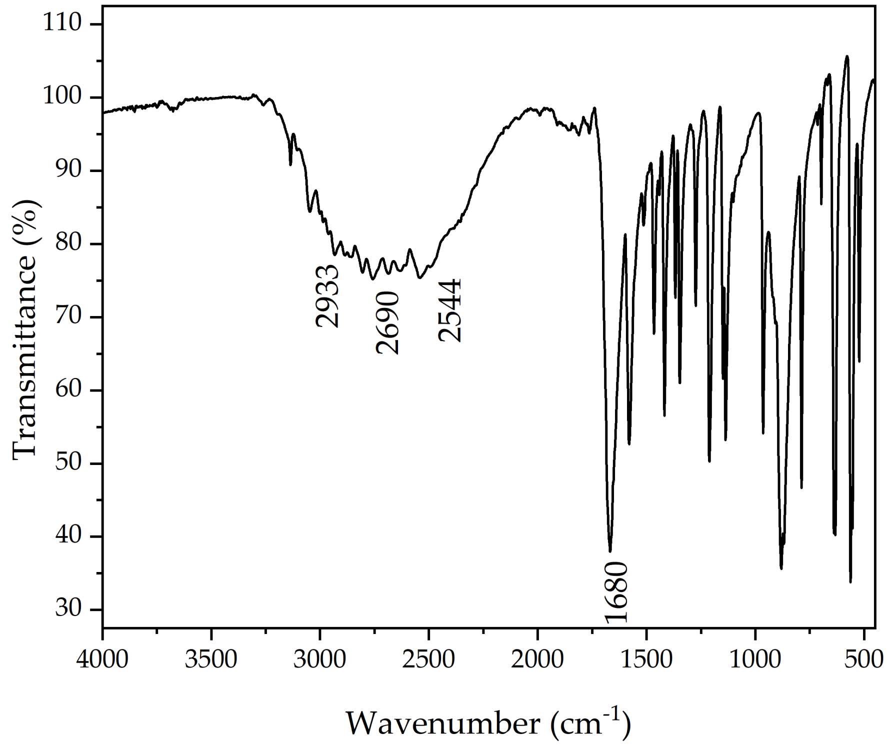

Figure S2. UATR-FTIR spectrum of hypoxanthine.

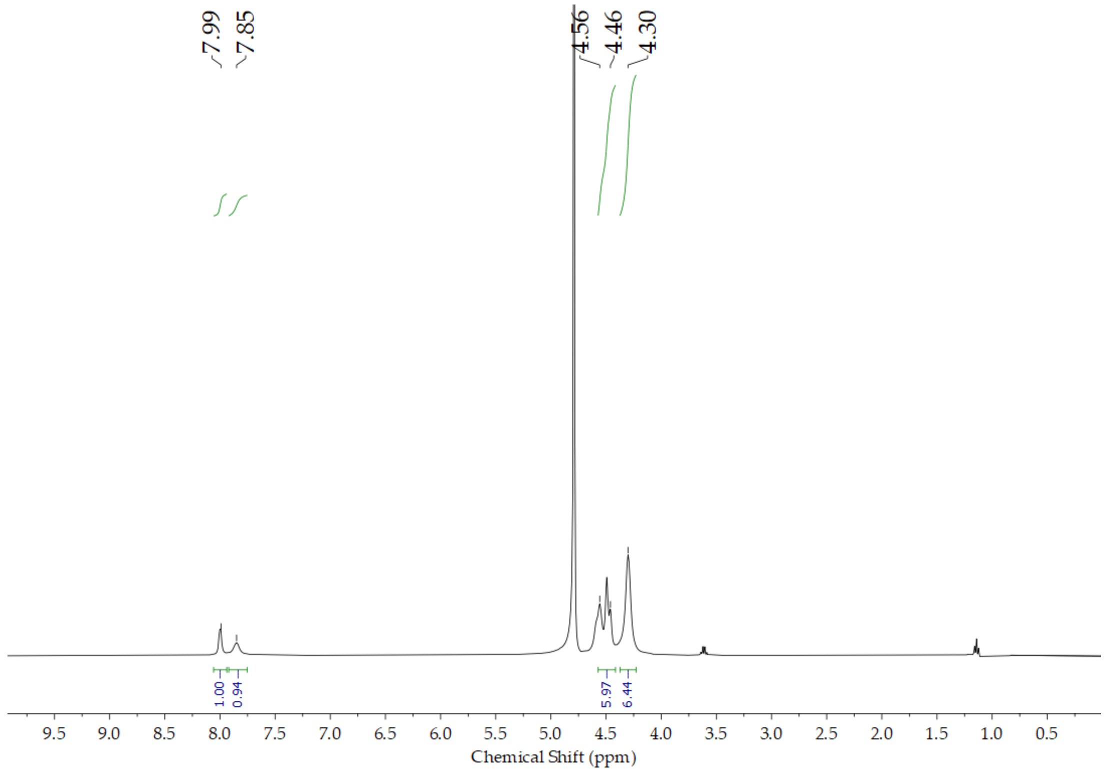

Figure S3. ^{1}H NMR (400 MHz, D_2O) spectrum of complex **1**.

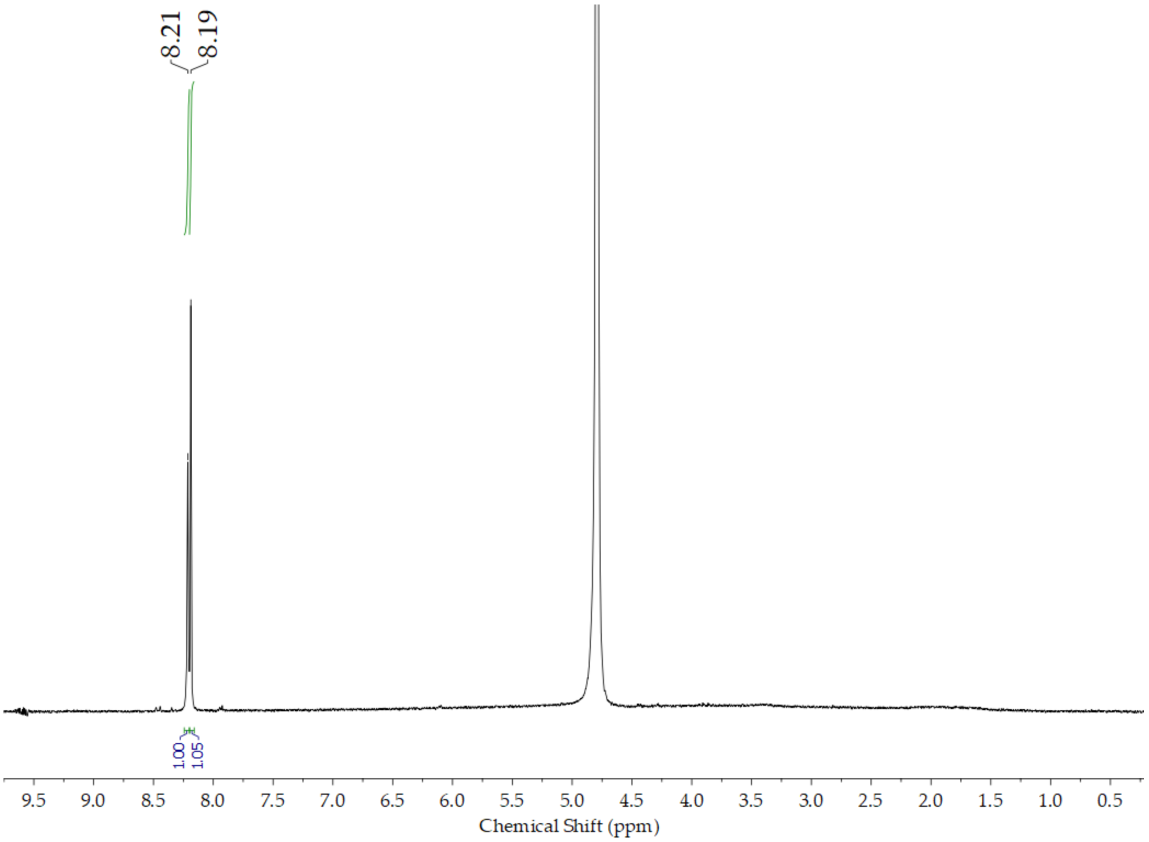

Figure S4. ^{1}H NMR (400 MHz, D_2O) spectrum of hypoxanthine.

molecules

MDPI

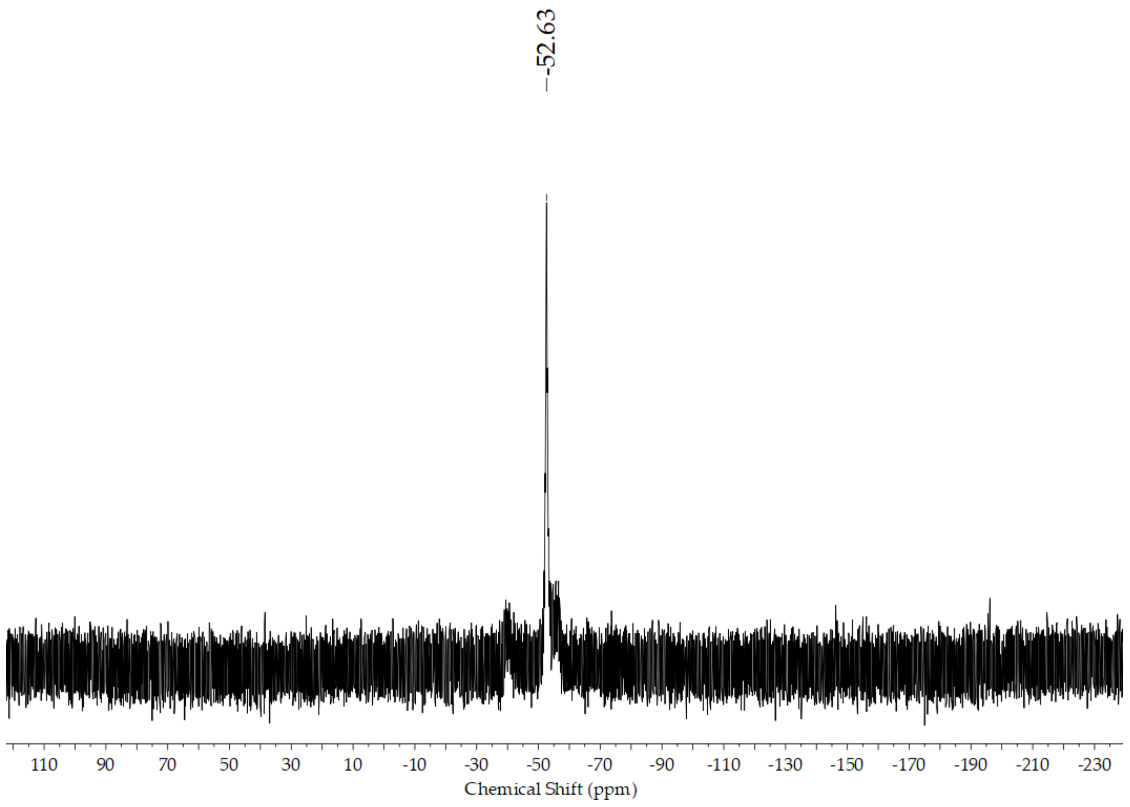

Figure S5. $^{31}P\{^{1}H\}$ NMR (162 MHz, D_2O) spectrum of complex **1**.

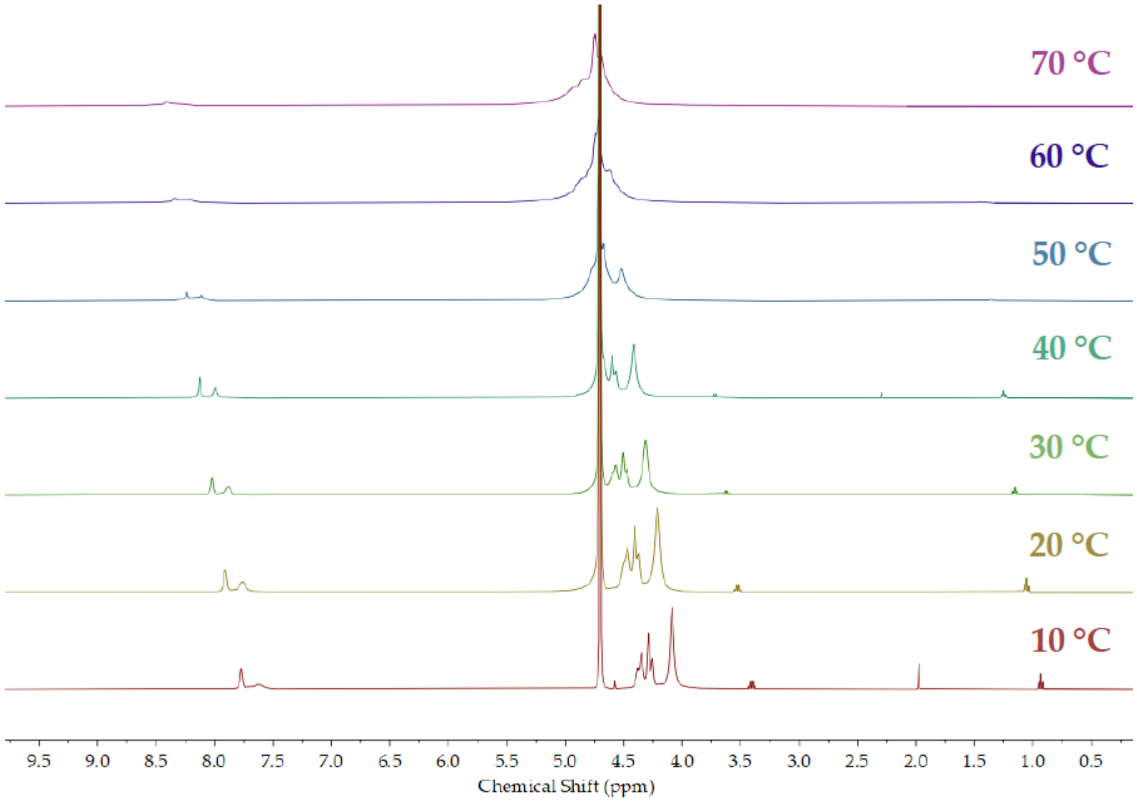

Figure S6. Collection of ^{1}H NMR spectra of complex **1** in D_2O (25 mM) at different temperatures.

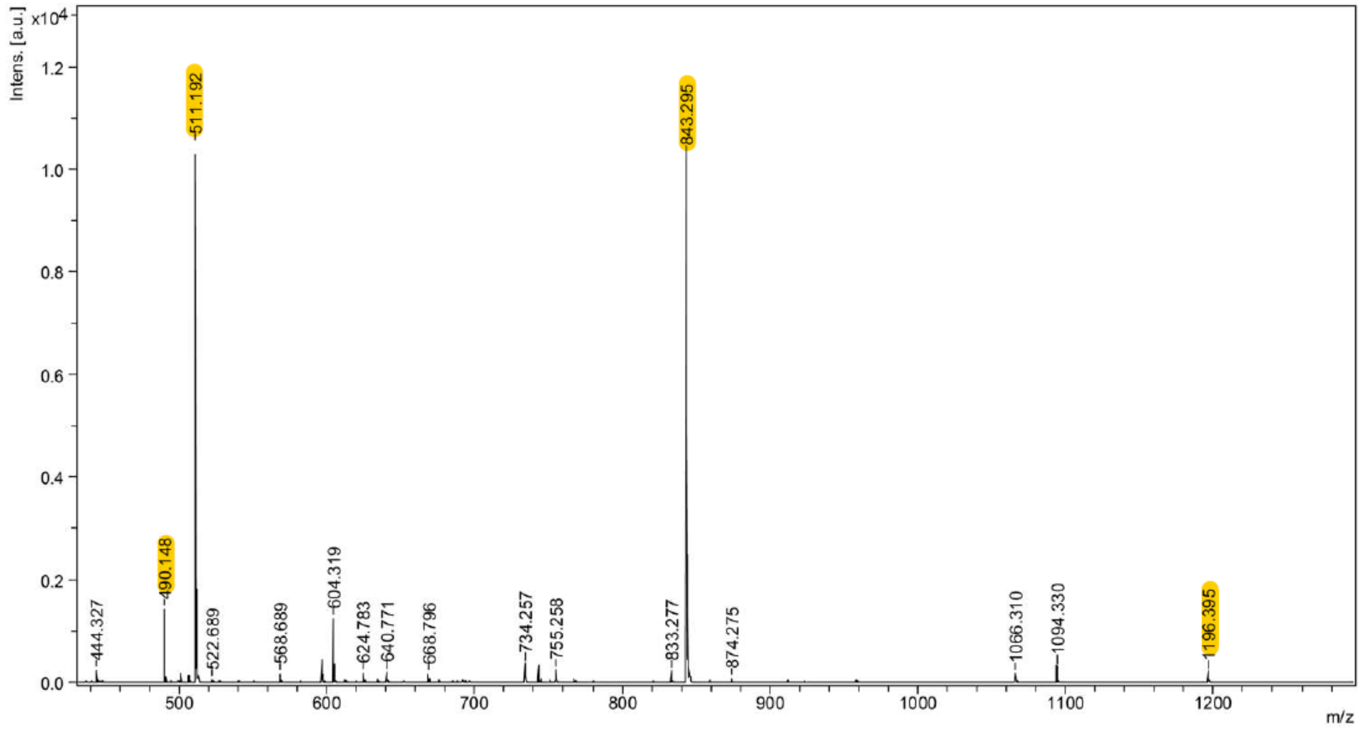

Figure S7. MALDI-MS(+) spectrum of complex **1**.

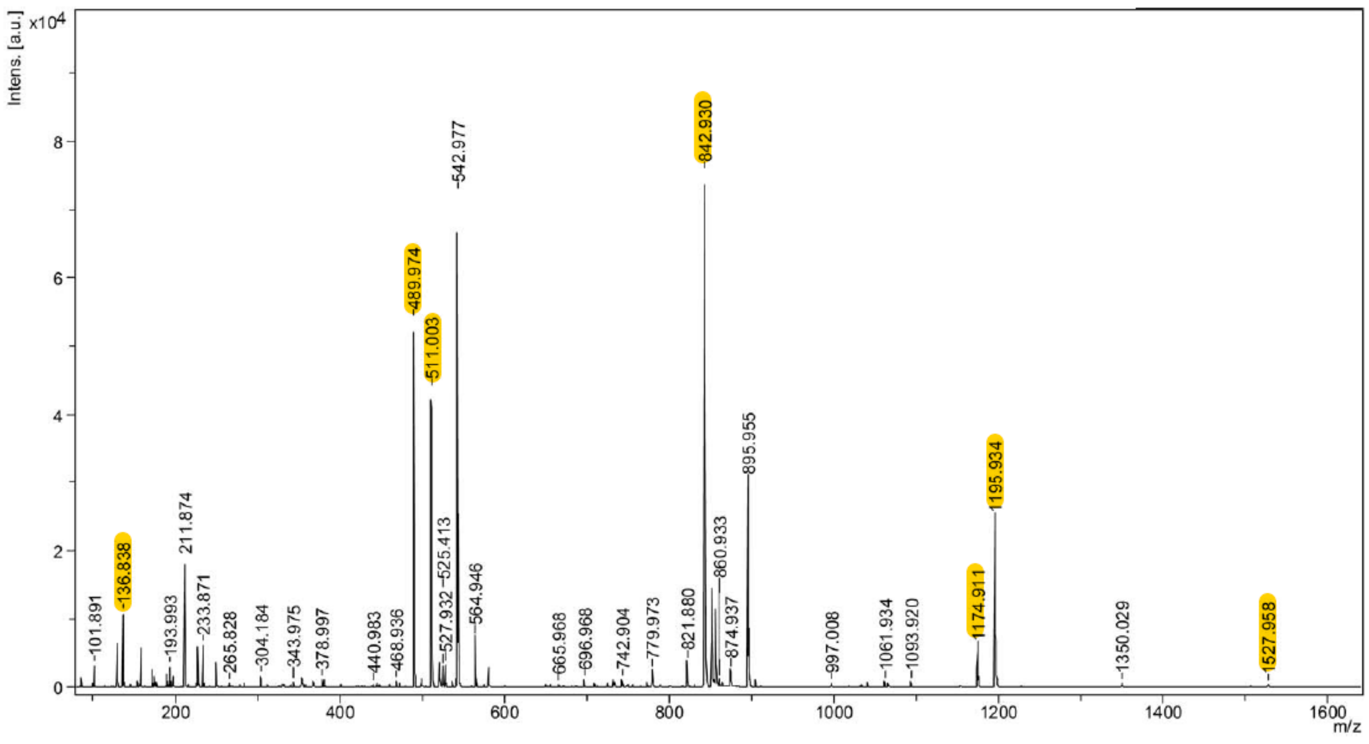

Figure S8. MALDI-MS(+) spectrum of an aged solution of complex **1**.

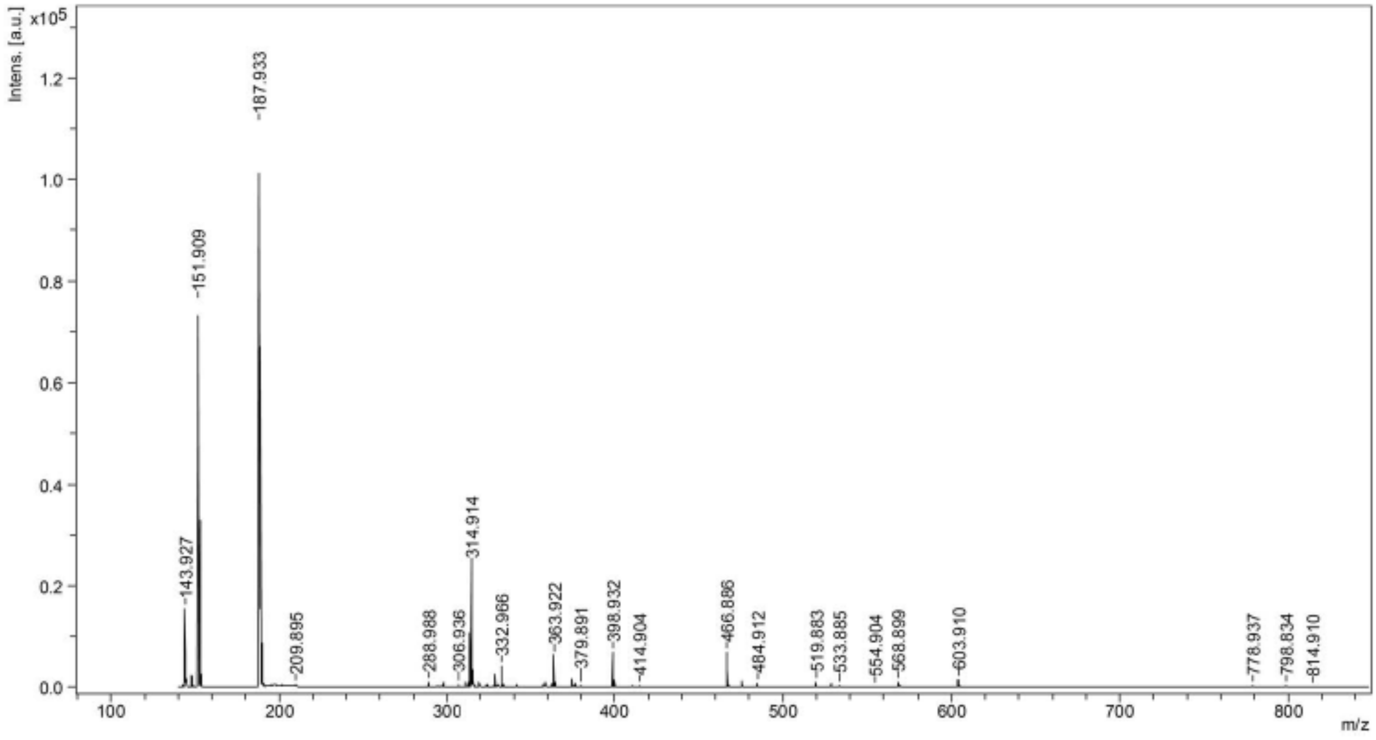

Figure S9. MALDI-MS(-) spectrum of complex **1**.

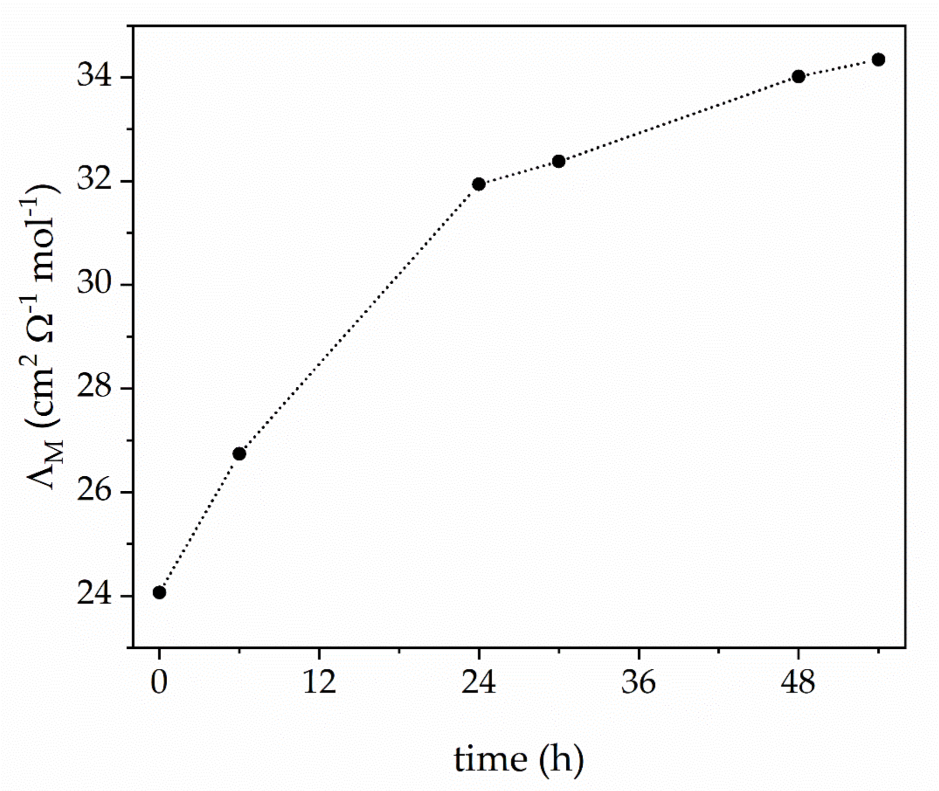

Figure S10. Molar conductivity in aqueous solution of complex **1** at different times.

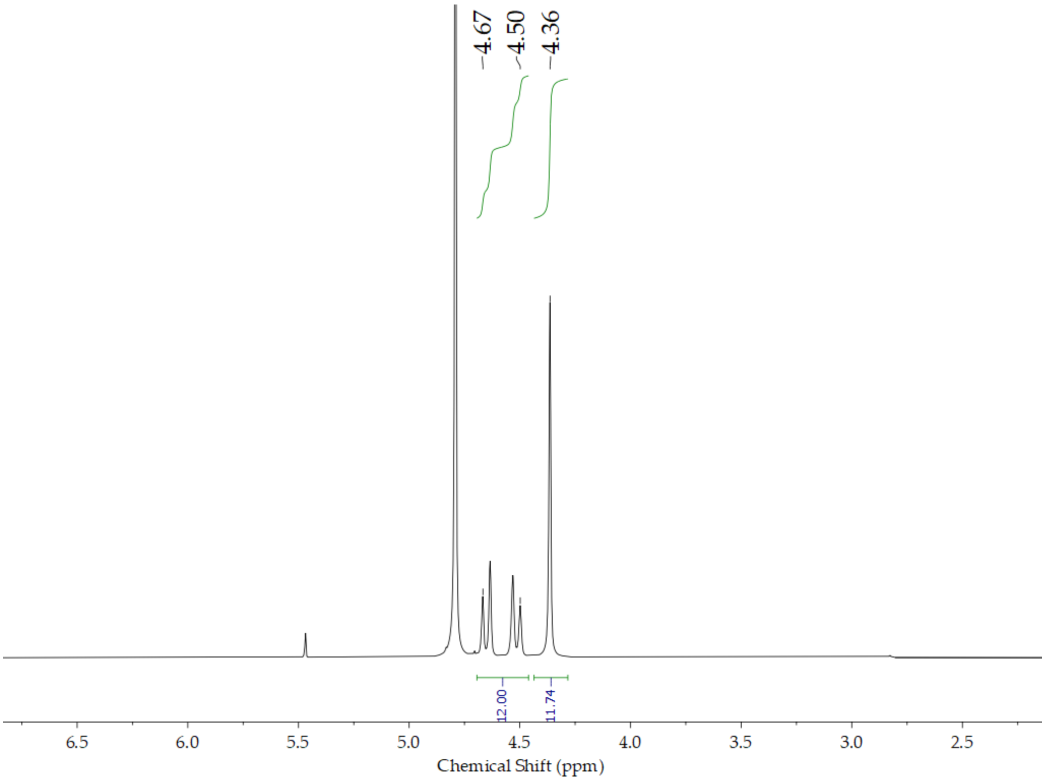

Figure S11. ^{1}H NMR (400 MHz, D_2O) spectrum of complex **2**.

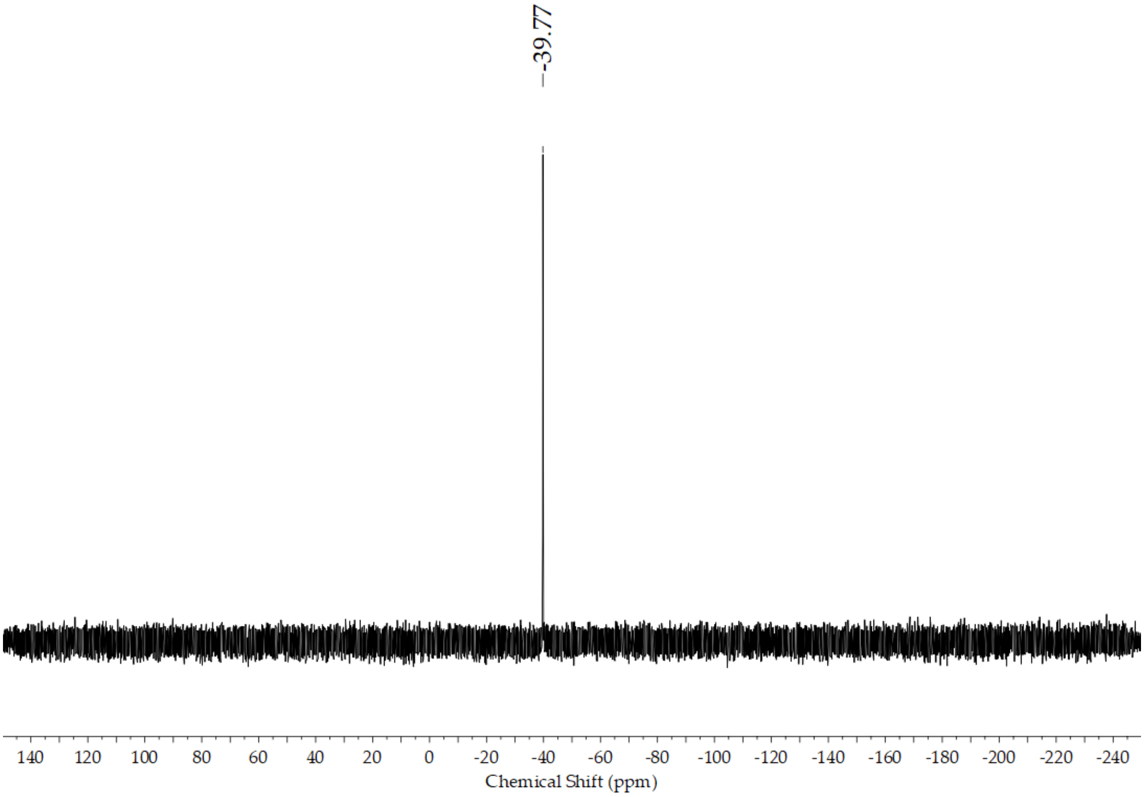

Figure S12. ^{31}P{^{1}H} NMR (162 MHz, D_2O) spectrum of complex **2**.

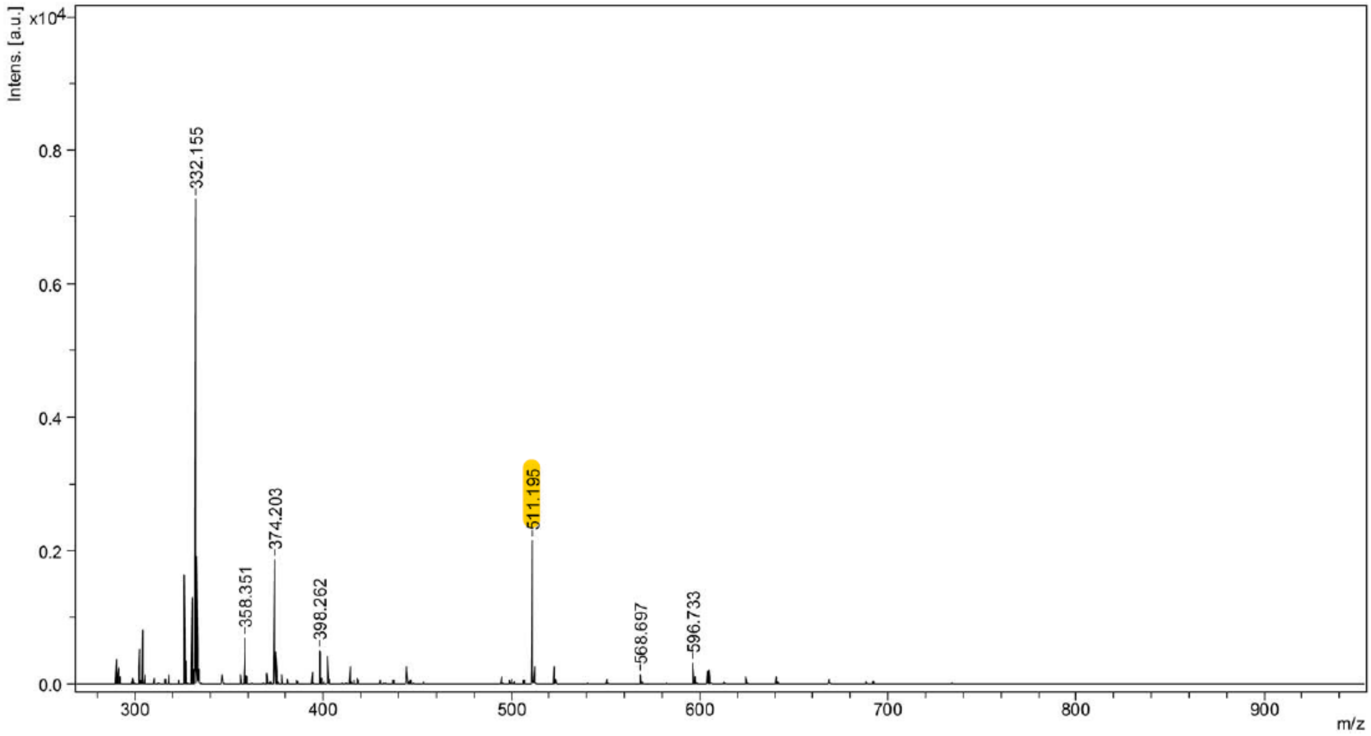

Figure S13. MALDI-MS(+) spectrum of complex **2**.

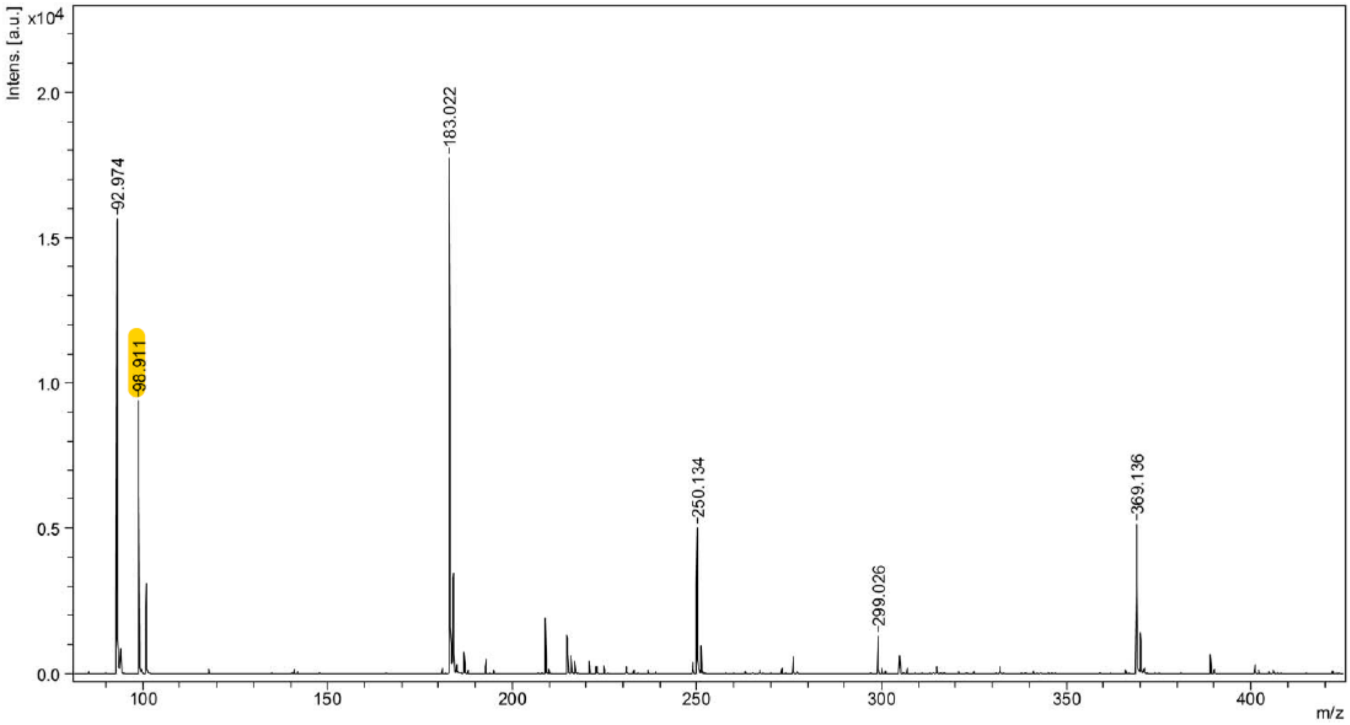

Figure S14. MALDI-MS(-) spectrum of complex **2**.

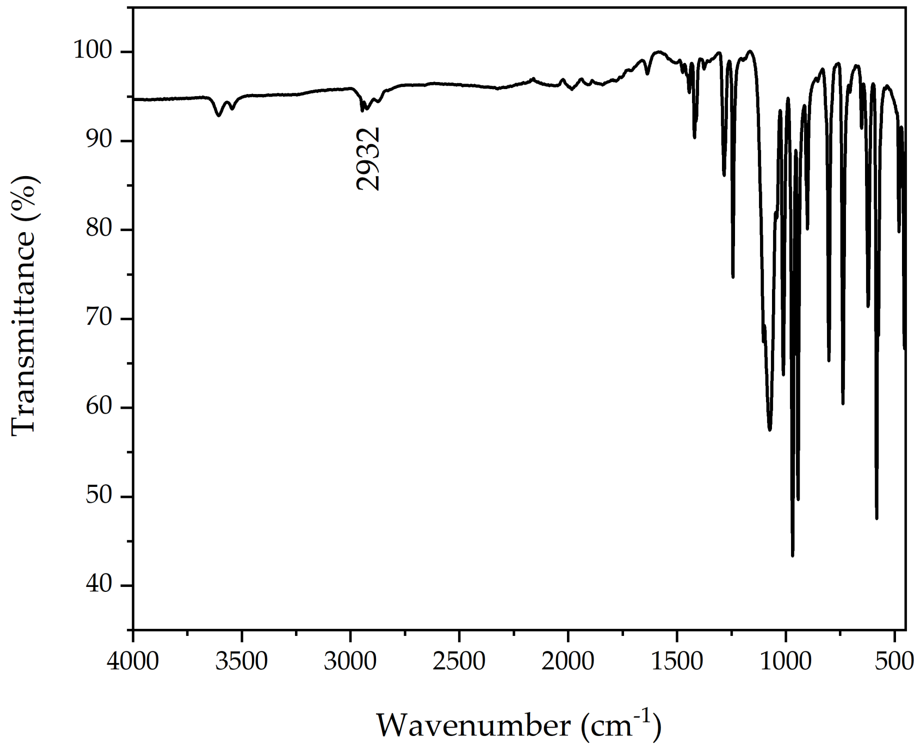

Figure S15. UATR-FTIR spectrum of complex **2**.

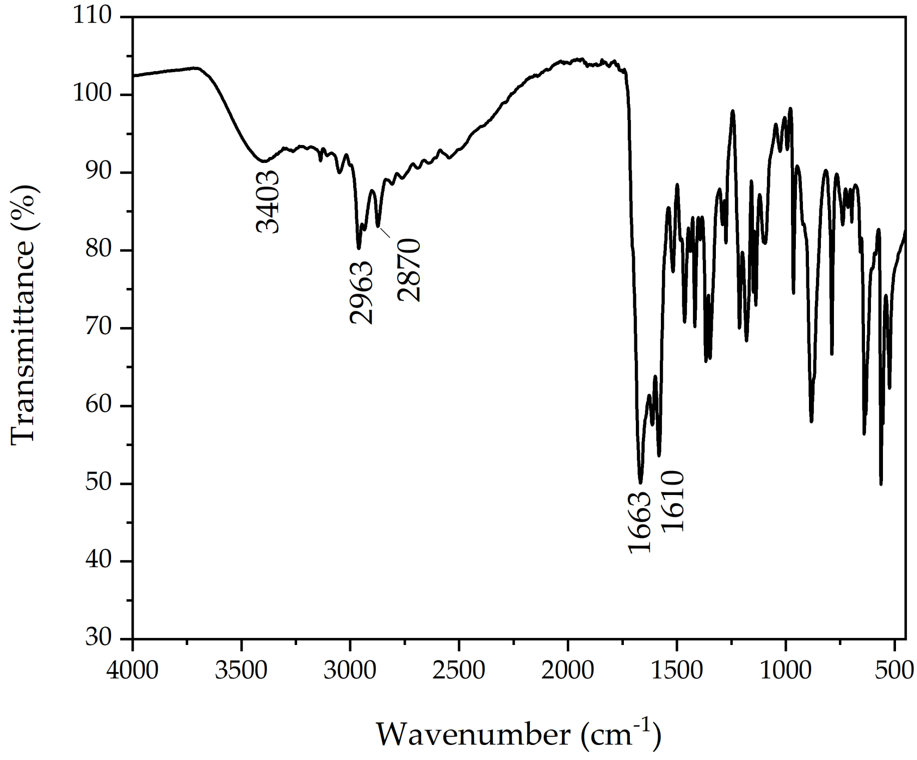

Figure S16. UATR-FTIR spectrum of complex **3**.

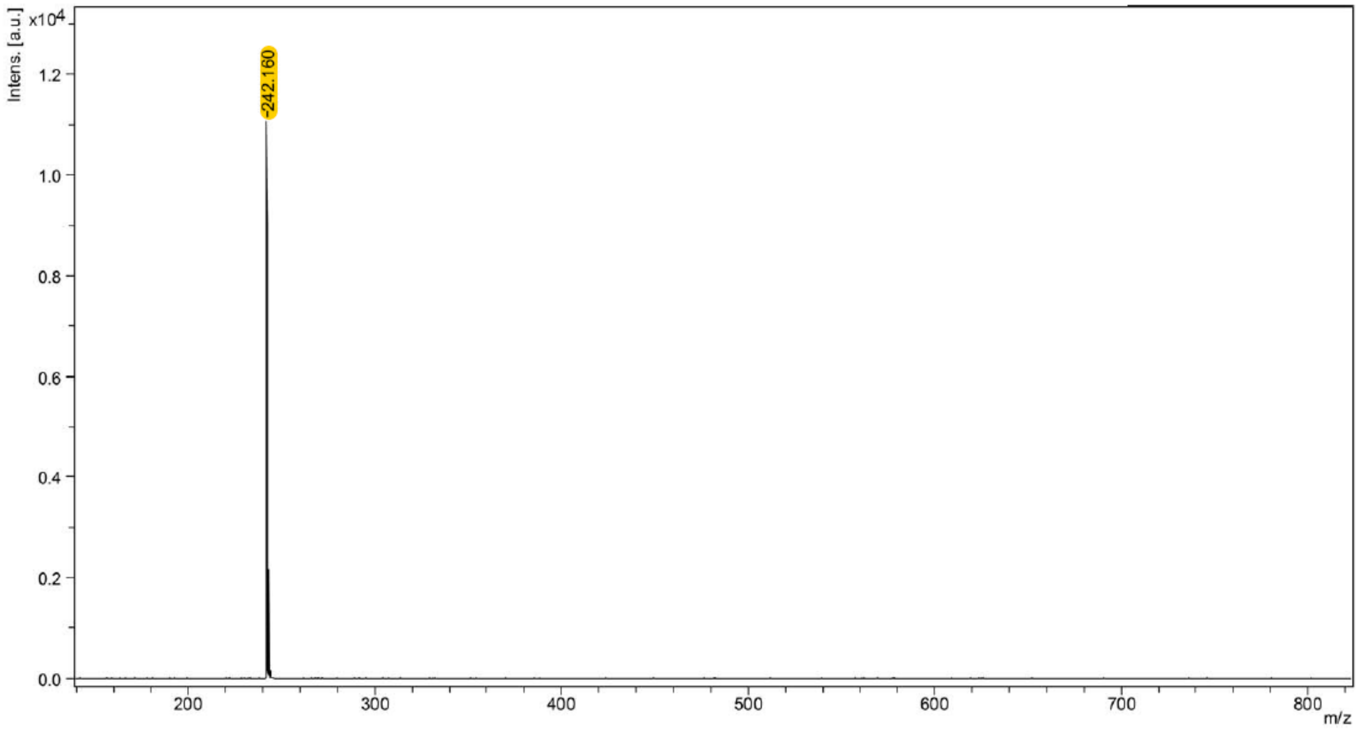

Figure S17. MALDI-MS(+) spectrum of complex **3**.

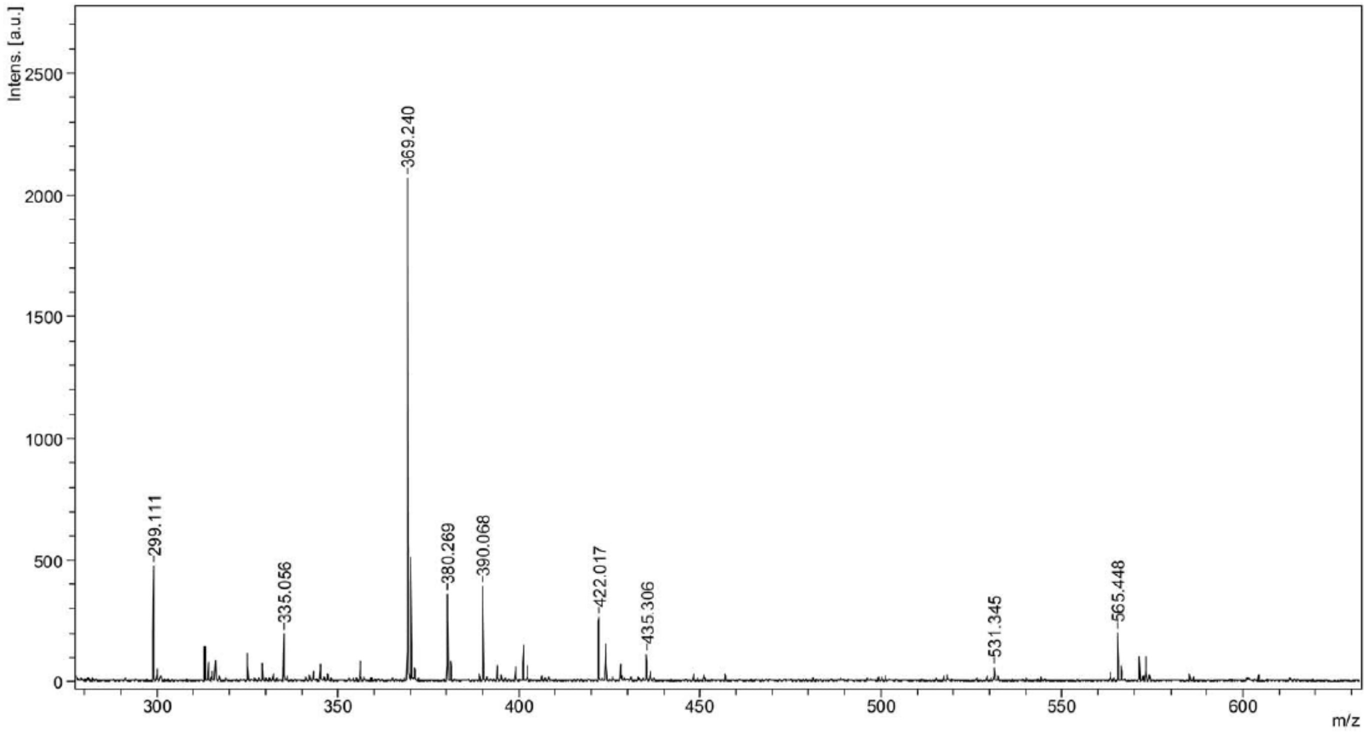

Figure S18. MALDI-MS(-) spectrum of complex **3**.

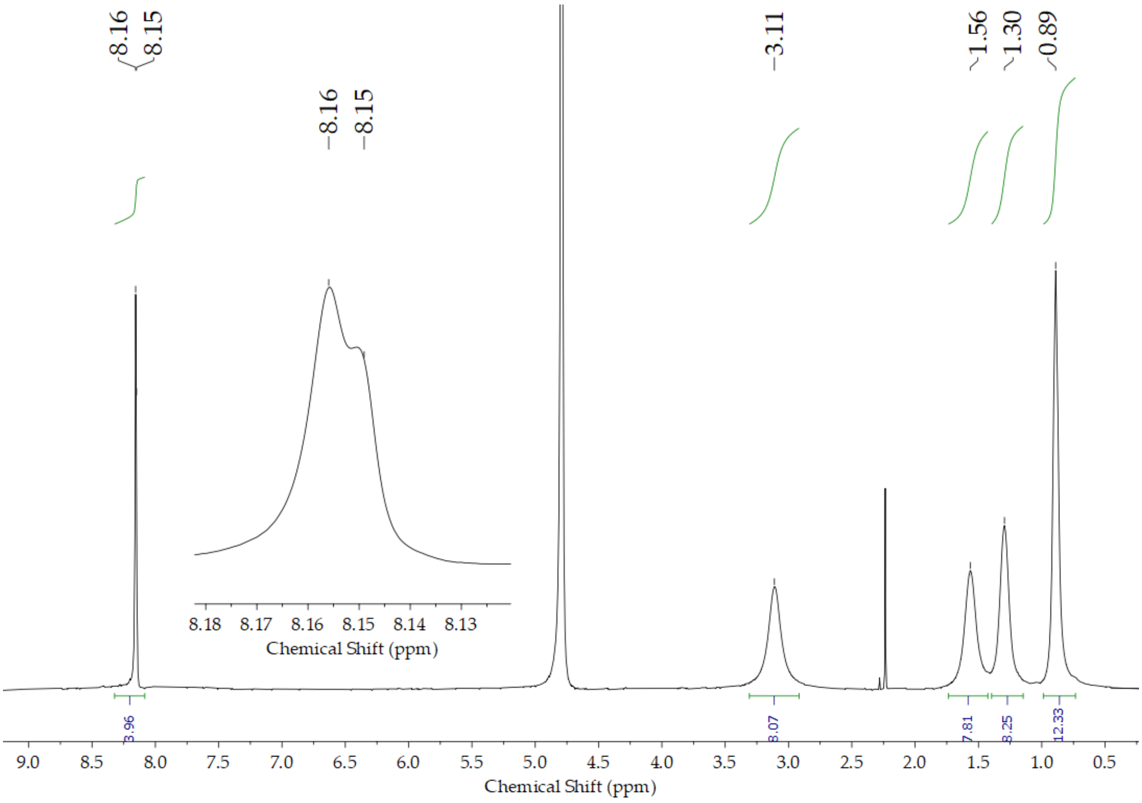

Figure S19. ^1H NMR (400 MHz, D_2O) spectrum of complex **3**.

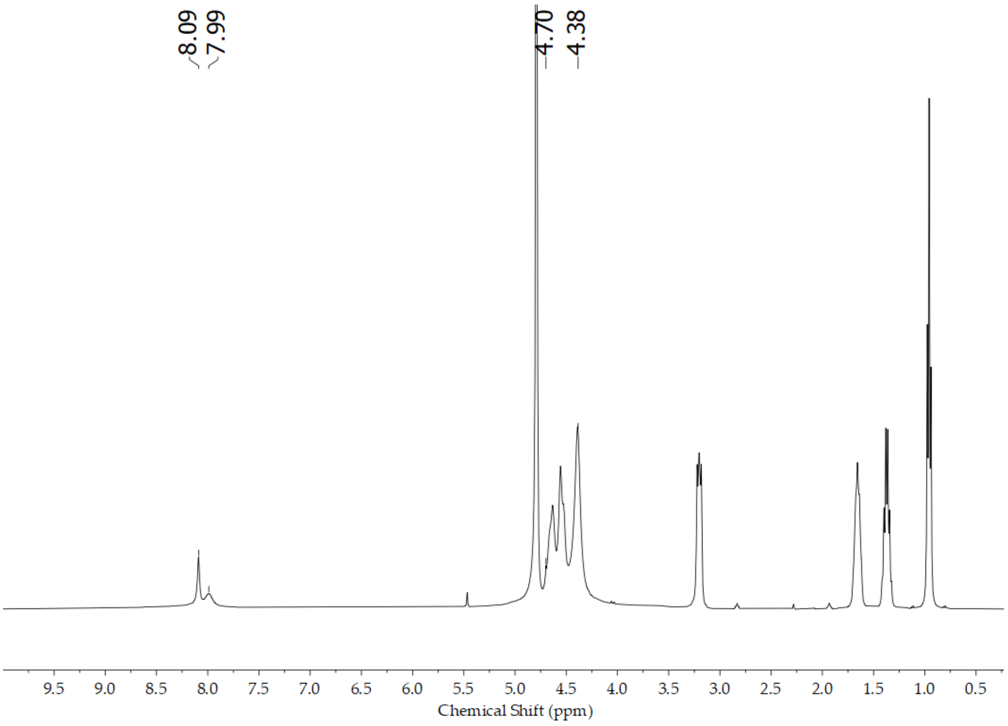

Figure S20. ^1H NMR (400 MHz, D_2O) spectrum of the equimolecular mixture of complexes **2** and **3**.

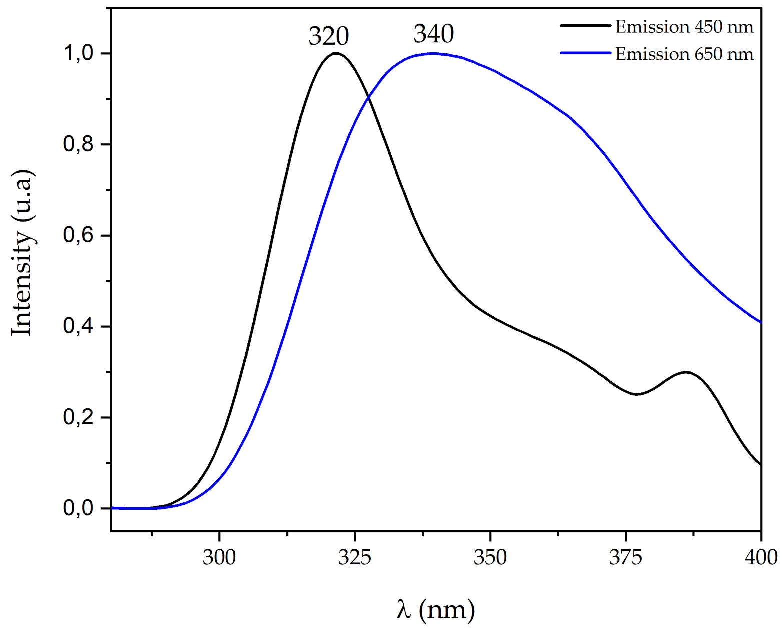

Figure S21. Excitation spectra of complex **1** in aqueous solution with an emission wavelength of 450 nm (black line) or 650 nm (blue line).

molecules

MDPI

$S_1 \leftarrow S_0$ (253 nm)

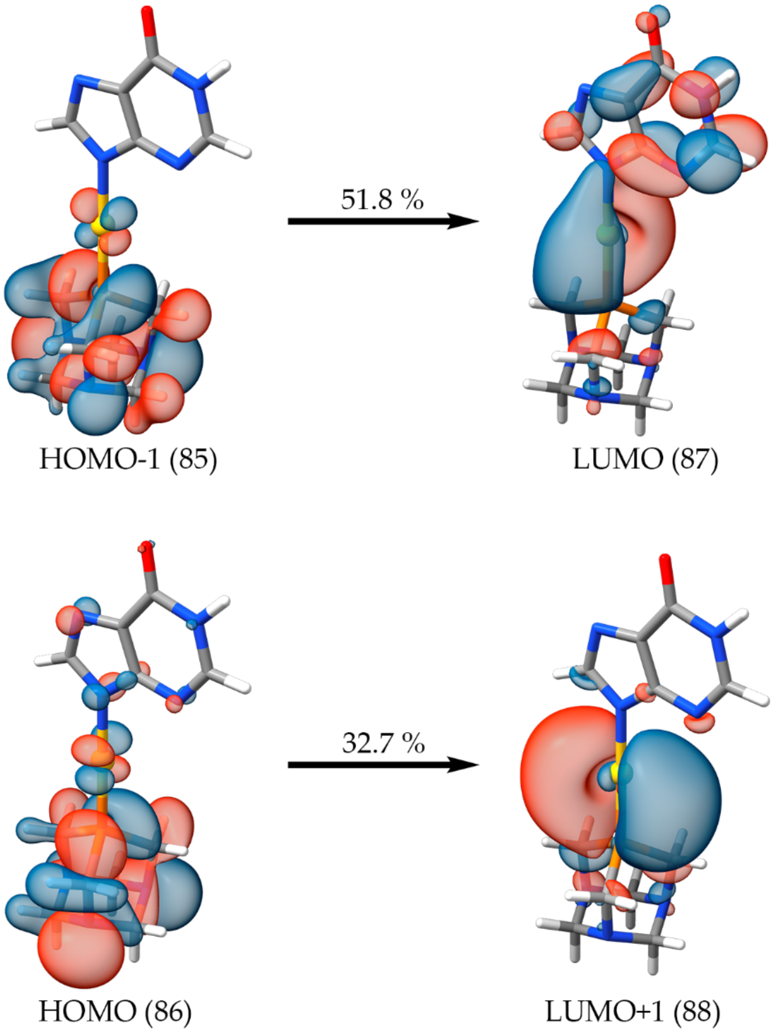

Figure S22. Molecular orbitals involved in the S_1 transition of model **1a**.

molecules MDPI

$S_5 \leftarrow S_0$ (247 nm)

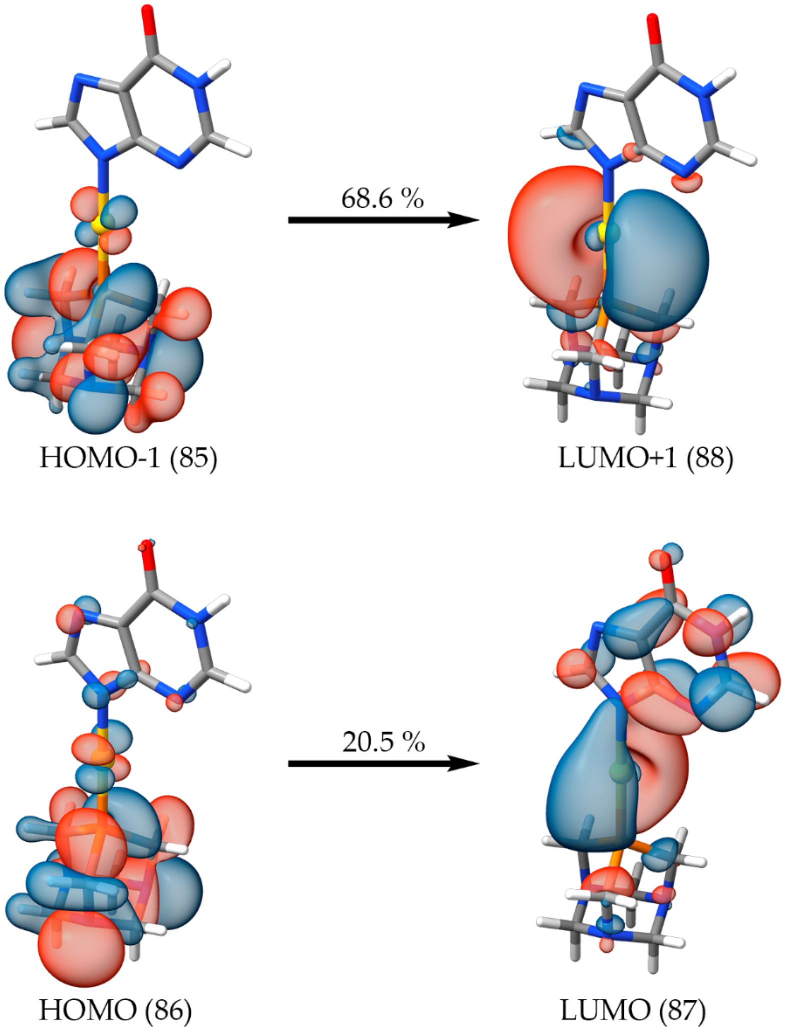

Figure S23. Molecular orbitals involved in the S_5 transition of model **1a**.

molecules MDPI

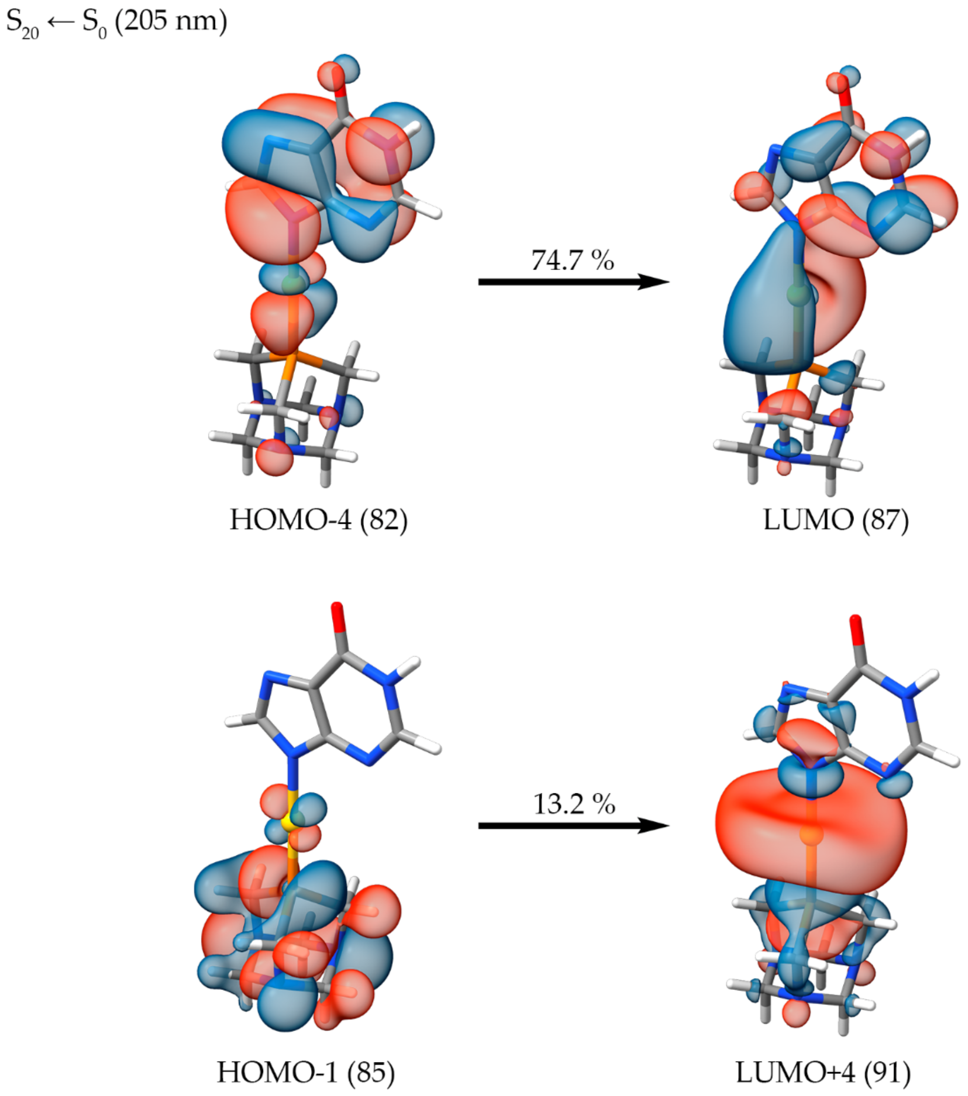

Figure S24. Molecular orbitals involved in the S_{20} transition of model **1a**.

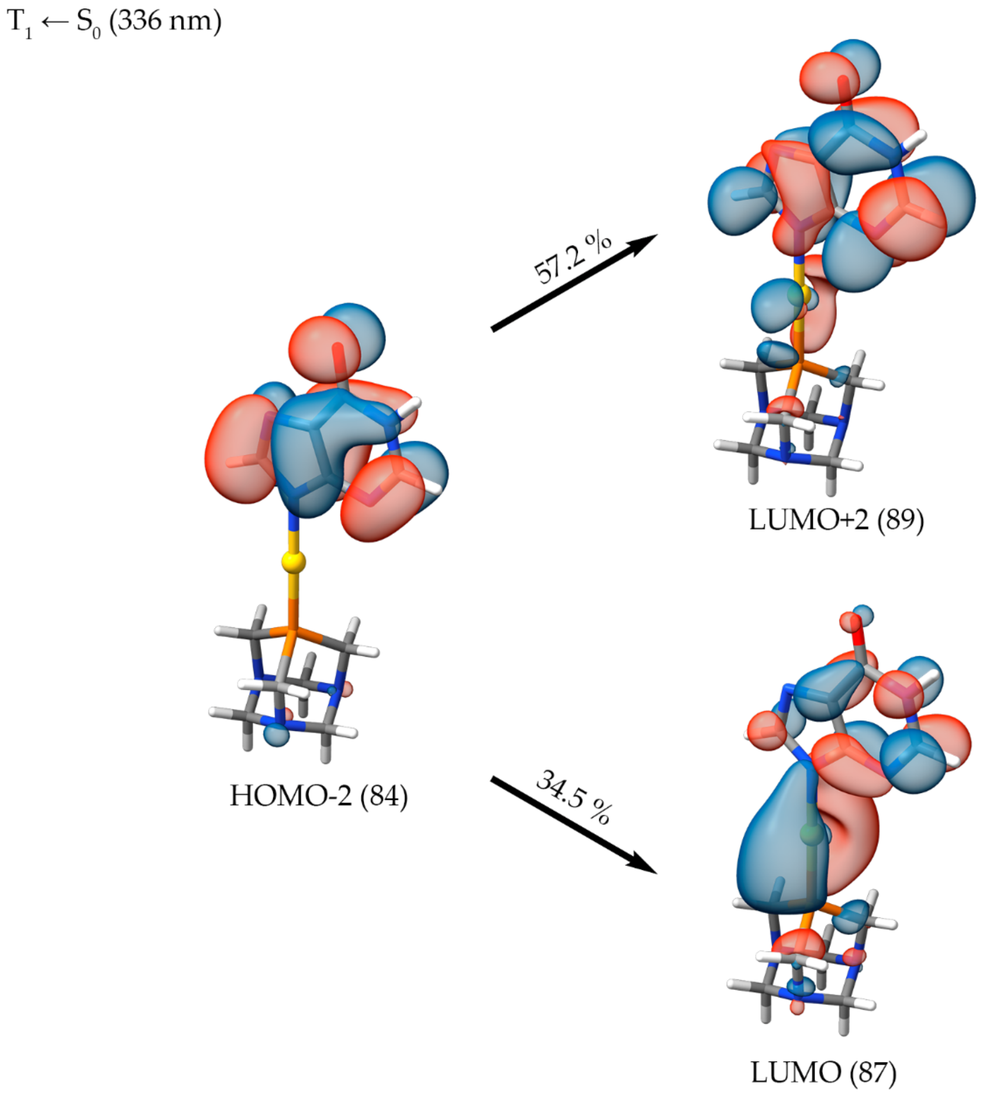

Figure S25. Molecular orbitals involved in the T_1 transition of model **1a**.

molecules MDPI

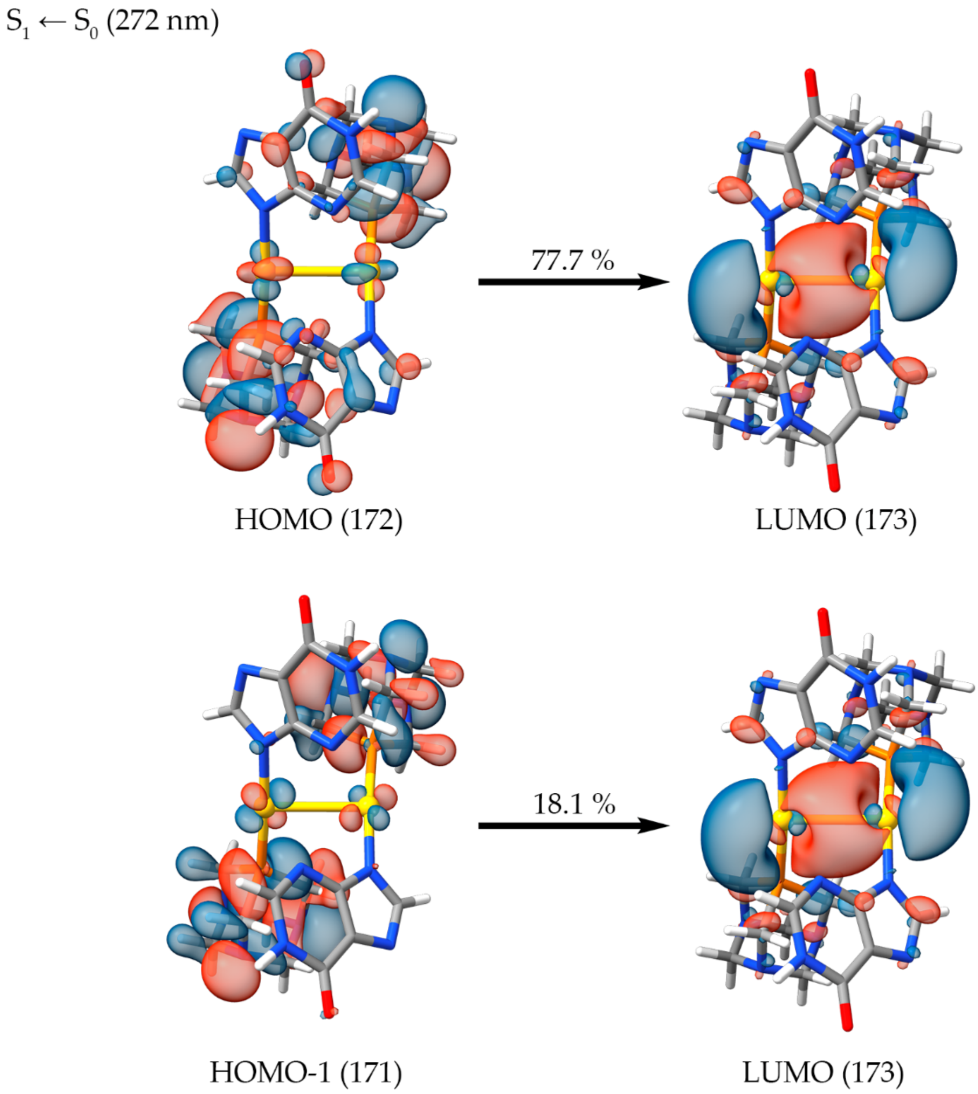

Figure S26. Molecular orbitals involved in the S_1 transition of model **1b**.

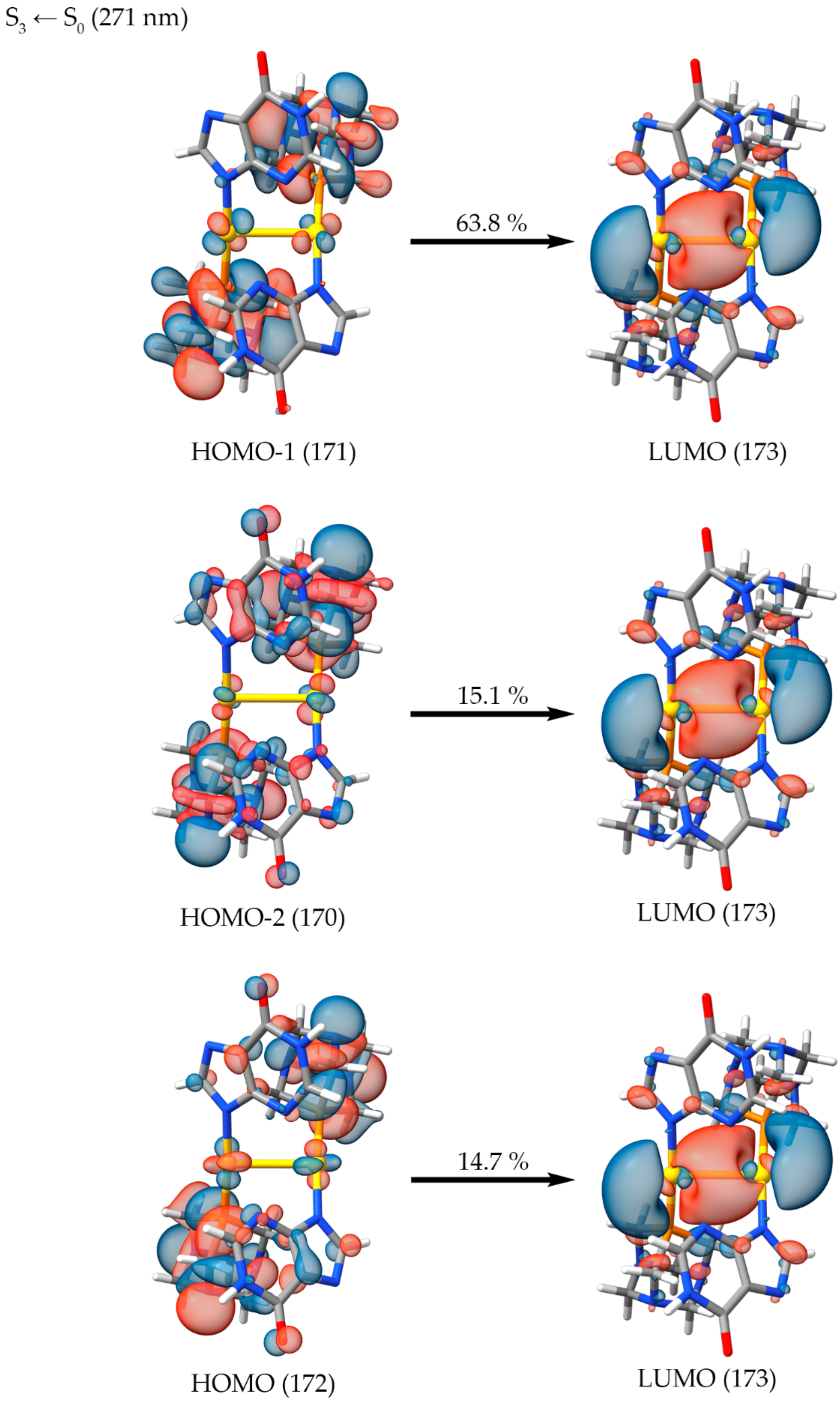

Figure S27. Molecular orbitals involved in the S_3 transition of model **1b**.

molecules

MDPI

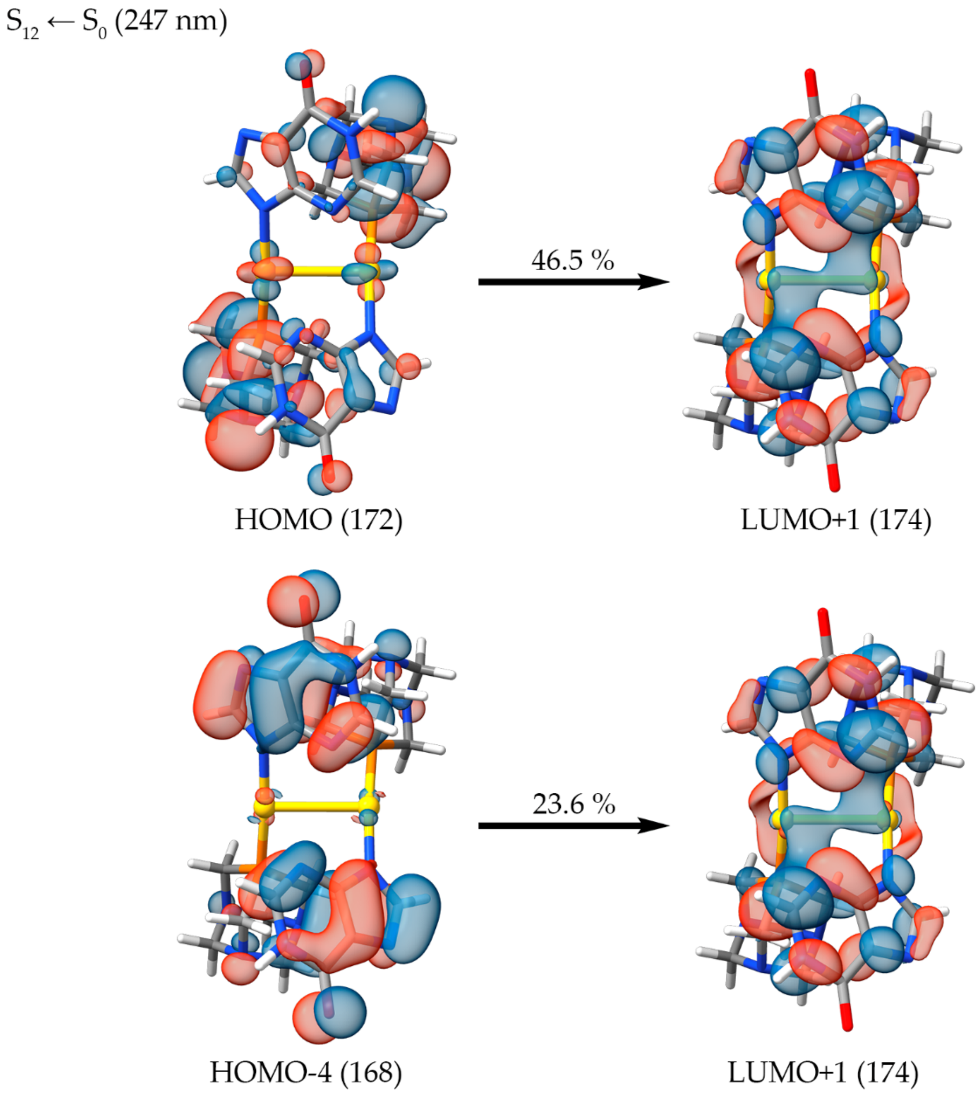

Figure S28. Molecular orbitals involved in the S_{12} transition of model **1b**.

molecules

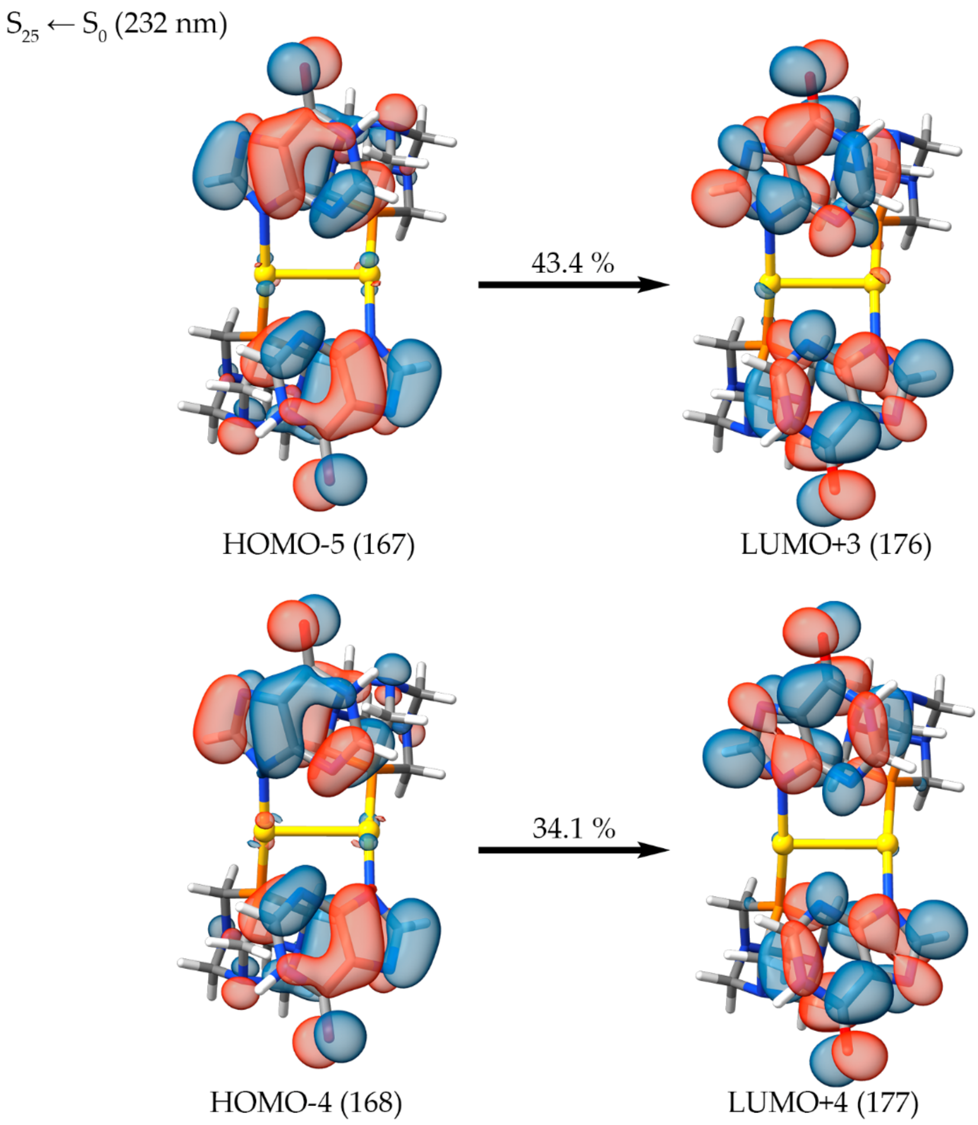

Figure S29. Molecular orbitals involved in the S_{25} transition of model **1b**.

molecules

MDPI

$T_1 \leftarrow S_0$ (337 nm)

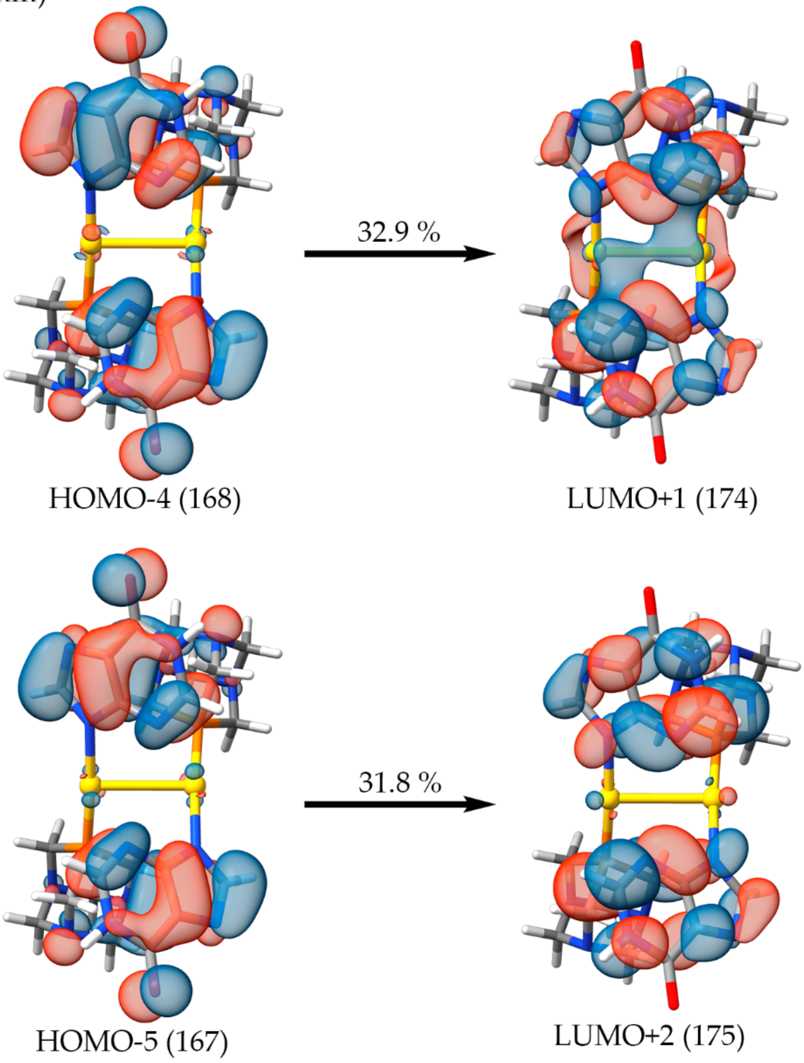

Figure S30. Molecular orbitals involved in the T_1 transition of model **1b**.

$S_1 \leftarrow S_0$ (288 nm)

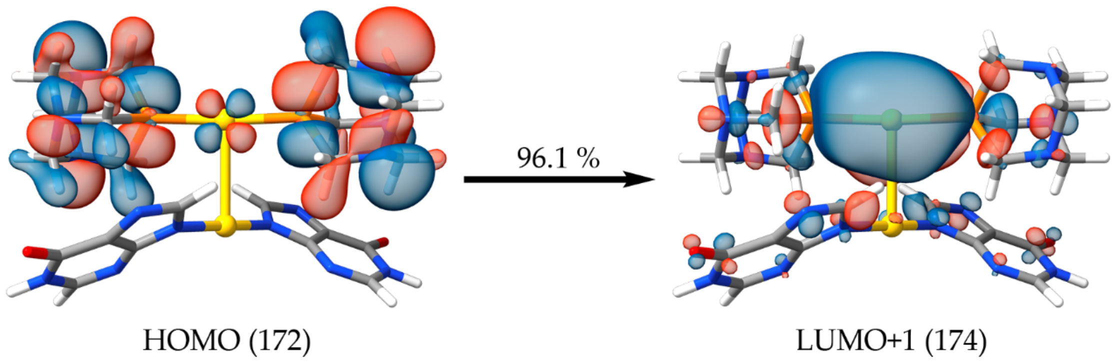

Figure S31. Molecular orbitals involved in the S_1 transition of model **1c**.

molecules

$S_4 \leftarrow S_0$ (281 nm)

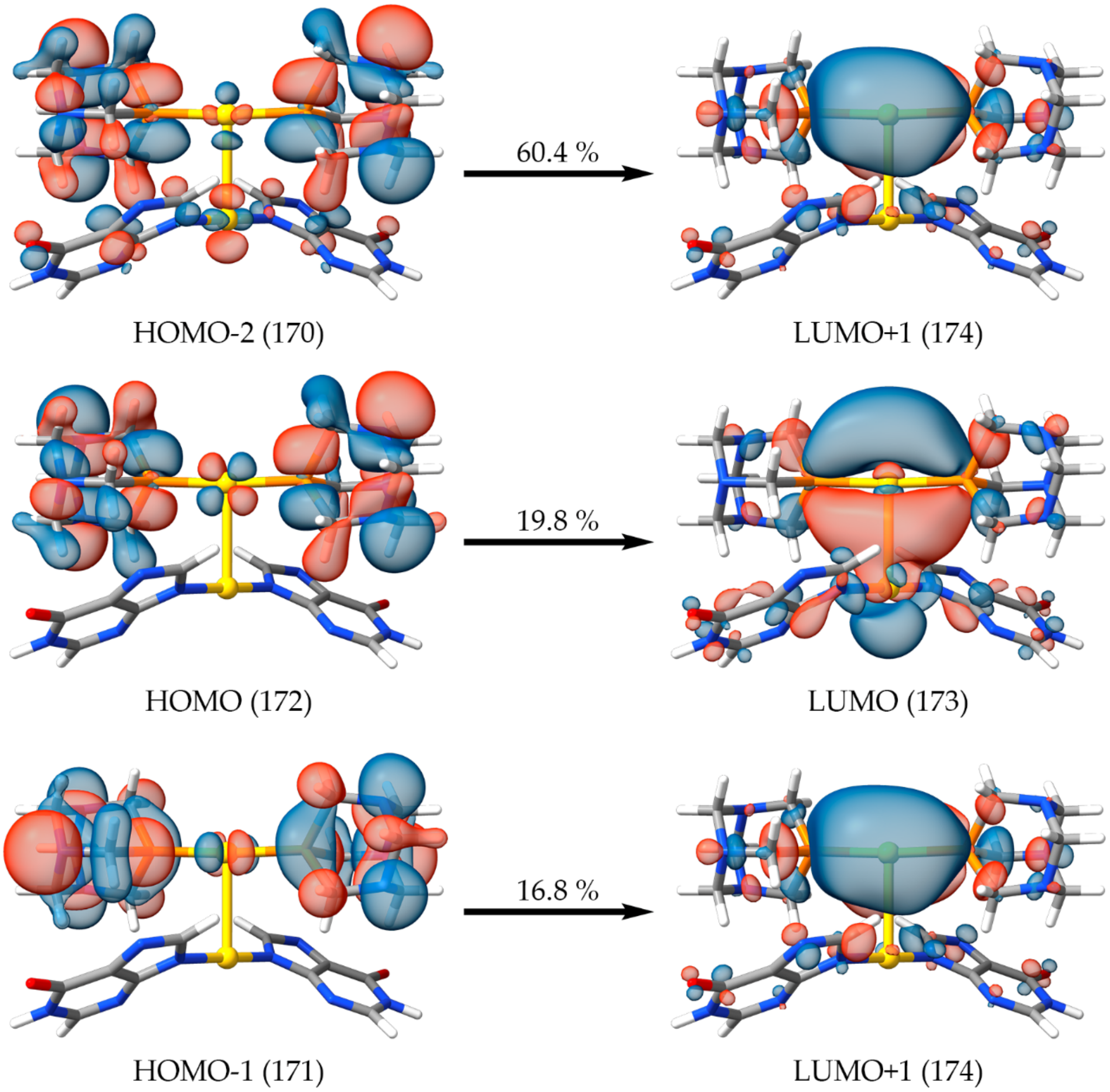

Figure S32. Molecular orbitals involved in the S_4 transition of model **1c**.

$S_5 \leftarrow S_0$ (281 nm)

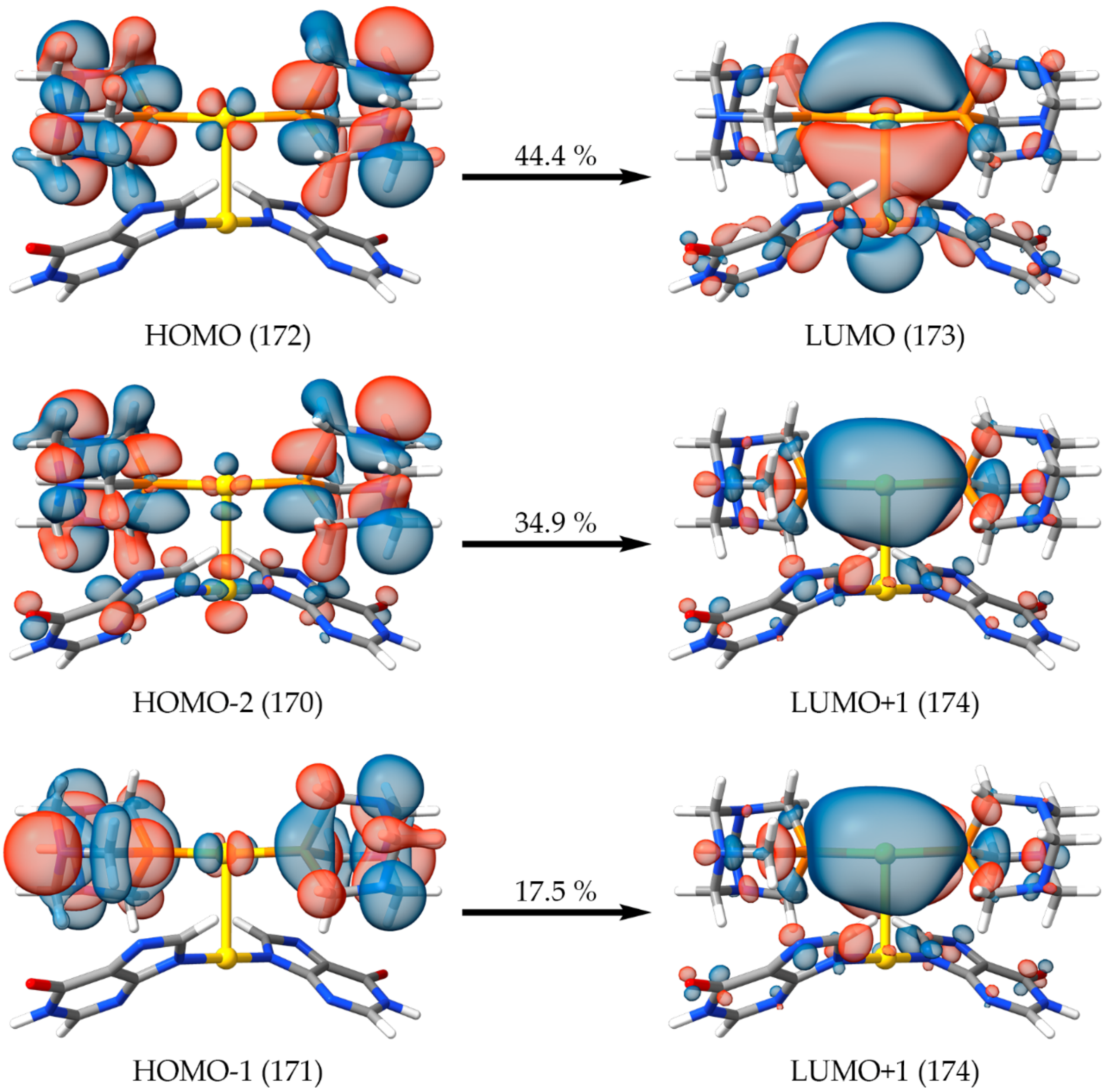

Figure S33. Molecular orbitals involved in the S_5 transition of model **1c**.

$S_{14} \leftarrow S_0$ (253 nm)

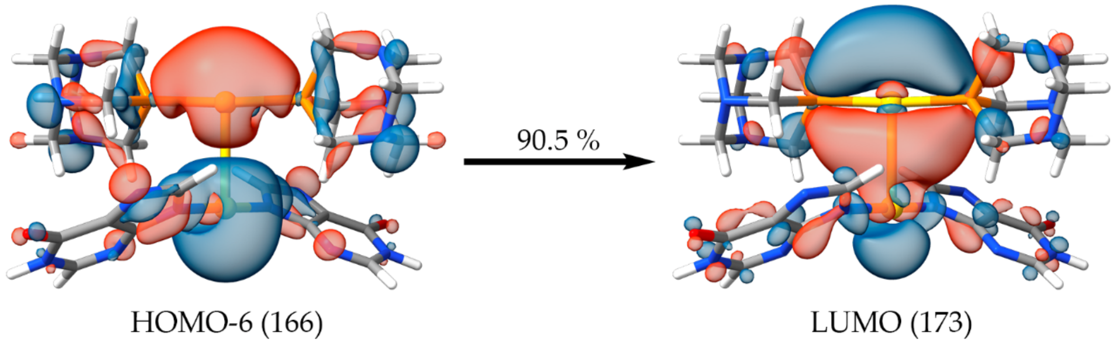

Figure S34. Molecular orbitals involved in the S_{14} transition of model **1c**.

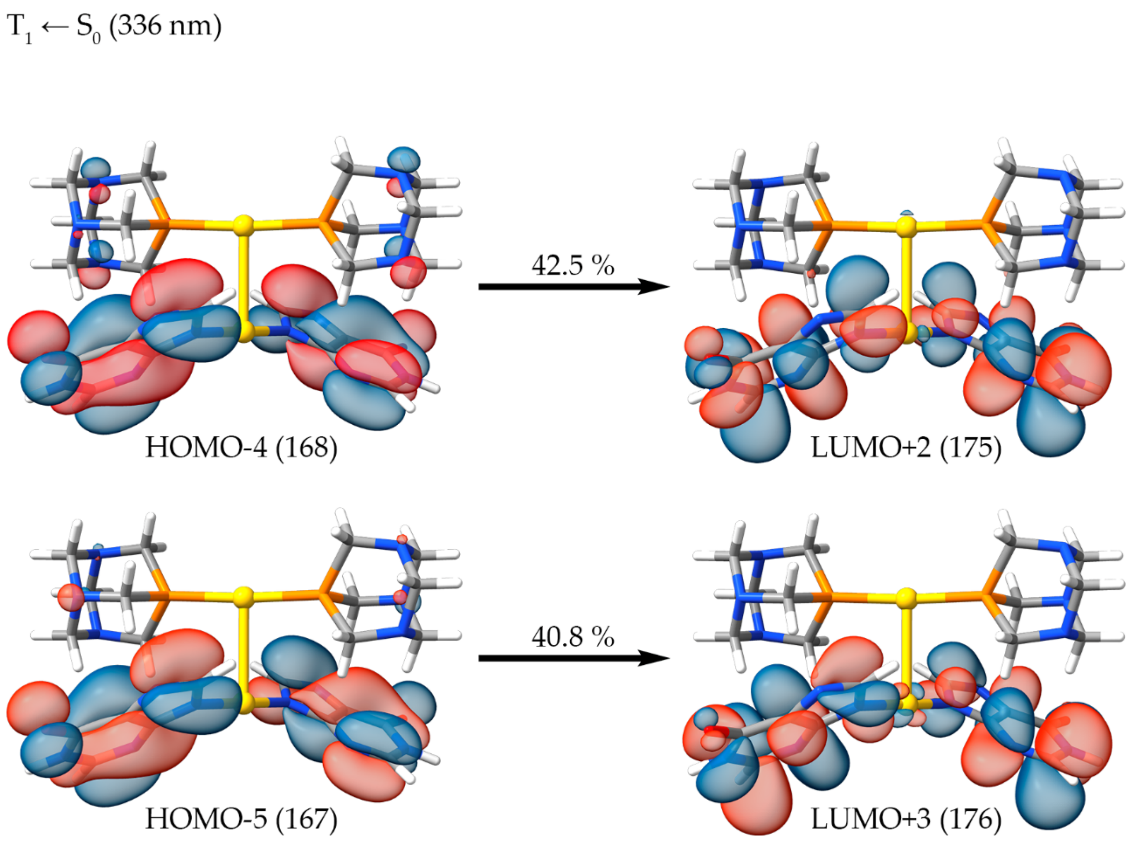

Figure S35. Molecular orbitals involved in the T_1 transition of model **1c**.

molecules

Table S1. Cartesian coordinates of the optimized structure [RI-DFT/PBE0-D3(BJ)/COSMO(ε = 80.1)/def2-TZVP/def2-ECP(Au)] of model **1a**.

36

N	-1.5915962	1.9520394	1.2485840
Au	-1.6721151	0.1340293	0.2999582
P	-1.7752921	-1.8450556	-0.7440931
C	-2.4549963	2.9994012	1.1449407
N	-2.1472729	4.0491255	1.8796385
C	-0.9966948	3.6675987	2.5151362
C	-0.6494541	2.3741310	2.1291104
C	-0.1769825	4.3688779	3.4452135
N	0.9060334	3.5750549	3.8304476
C	1.1630001	2.3178738	3.3975463
N	0.4281756	1.6664973	2.5525450
C	-2.9758421	-2.0416448	-2.1282654
C	-2.2160202	-3.3421740	0.2359024
C	-0.2620478	-2.4821617	-1.5808361
N	-2.8973445	-3.3868913	-2.6805138
H	-2.7519893	-1.3035640	-2.9017520
H	-3.9839993	-1.8456400	-1.7563319
C	-1.5723772	-3.6725373	-3.2211680
N	-0.5313745	-3.7710094	-2.2033044
H	-1.3014732	-2.8945380	-3.9380502
H	-1.6224387	-4.6305538	-3.7441231
H	0.5385405	-2.5799140	-0.8441764
H	0.0593111	-1.7597084	-2.3345033
C	-3.2261071	-4.4004726	-1.6834980
N	-2.2348794	-4.5209284	-0.6193496
H	-3.2903154	-5.3646883	-2.1933440
H	-4.1988510	-4.1698383	-1.2439860
C	-0.9293081	-4.7734021	-1.2202957
H	-0.9739235	-5.7407511	-1.7263169
H	-0.1747690	-4.8233318	-0.4324268
H	-1.4860896	-3.4707529	1.0383197
H	-3.1968691	-3.1928390	0.6929310
H	1.5466273	3.9909971	4.4928108
O	-0.3132866	5.5008604	3.9028734
H	2.0543695	1.8586553	3.8097336
H	-3.3224142	2.9512153	0.5012693

molecules

MDPI

Table S2. Cartesian coordinates of the optimized structure [RI-DFT/PBE0-D3(BJ)/COSMO(ε = 80.1)/def2-TZVP/def2-ECP(Au)] of model **1b**.

72

N	-1.5531090	1.8426882	1.4003318
Au	-1.5573891	0.0024533	0.4652486
P	-1.7239738	-1.9173100	-0.6802049
C	-2.4154385	2.8703267	1.1651517
N	-2.1259497	3.9960164	1.7873819
C	-0.9889897	3.6914418	2.4842265
C	-0.6308455	2.3625329	2.2513717
C	-0.1892222	4.4988983	3.3452151
N	0.8966188	3.7640243	3.8268846
C	1.1683773	2.4673165	3.5374136
N	0.4478701	1.7208974	2.7641504
C	-2.9400192	-2.0025460	-2.0631981
C	-2.2081795	-3.4535155	0.2175460
C	-0.2432441	-2.5690829	-1.5670087
N	-2.9155947	-3.3172470	-2.6882365
H	-2.6972999	-1.2317561	-2.7986722
H	-3.9371900	-1.7900951	-1.6712943
C	-1.6073256	-3.6199867	-3.2580132
N	-0.5620899	-3.8091757	-2.2574940
H	-1.3144058	-2.8143378	-3.9345728
H	-1.6956319	-4.5459434	-3.8310346
H	0.5544568	-2.7415905	-0.8419208
H	0.1087051	-1.8180396	-2.2787753
C	-3.2717897	-4.3707761	-1.7439821
N	-2.2760743	-4.5825276	-0.6980128
H	-3.3744222	-5.3035199	-2.3034687
H	-4.2315590	-4.1303250	-1.2819531
C	-0.9855496	-4.8478936	-1.3245344
H	-1.0668482	-5.7858706	-1.8785186
H	-0.2249370	-4.9651745	-0.5484108
H	-1.4714156	-3.6541243	0.9980588
H	-3.1764940	-3.2970701	0.6978989
H	1.5261708	4.2570571	4.4457763
O	-0.3422183	5.6727564	3.6712473
H	2.0623972	2.0661621	4.0013379
H	-3.2674564	2.7492970	0.5106910
Au	1.5584467	-0.0030791	0.4648044
P	1.7245160	1.9168061	-0.6805007
N	1.5539537	-1.8430849	1.4002697
C	2.9408824	2.0033180	-2.0631461
C	0.2435260	2.5678239	-1.5674463
C	2.2074279	3.4531093	0.2177923
N	2.2746951	4.5824476	-0.6973928
H	1.4703844	3.6529307	0.9982313
H	3.1758014	3.2971577	0.6981621
N	2.9156048	3.3181885	-2.6877868
N	0.5616311	3.8083219	-2.2575170
H	-0.5542369	2.7395608	-0.8422375
H	-0.1078020	1.8167328	-2.2794698
C	1.6072735	3.6201421	-3.2578226

C	0.9841359	4.8470598	-1.3241704
H	0.2232656	4.9635351	-0.5481694
H	1.0648659	5.7852745	-1.8778268
C	3.2708192	4.3717404	-1.7431815
H	1.6950707	4.5463005	-3.8305900
H	1.3151208	2.8144484	-3.9346590
H	2.6989238	1.2325497	-2.7989008
H	3.9381448	1.7914809	-1.6711317
H	4.2306905	4.1319292	-1.2810239
H	3.3728536	5.3046995	-2.3024162
C	2.4160801	-2.8709732	1.1653679
N	2.1262207	-3.9964959	1.7877232
C	0.9892631	-3.6915334	2.4843999
C	0.6314183	-2.3625972	2.2511943
N	-0.4471663	-1.7205619	2.7637157
C	-1.1677735	-2.4664657	3.5373588
N	-0.8962504	-3.7631425	3.8272483
C	0.1893432	-4.4984885	3.3457142
O	0.3420071	-5.6722455	3.6723099
H	-1.5256015	-4.2556463	4.4467568
H	3.2682738	-2.7502211	0.5110815
H	-2.0617433	-2.0649827	4.0010873

Table S3. Cartesian coordinates of the optimized structure [RI-DFT/PBE0-D3(BJ)/COSMO(ε = 80.1)/def2-TZVP/def2-ECP(Au)] of model **1c**.

72

P	2.1392081	-0.8683558	-1.4014276
Au	0.0000000	-0.0000000	-1.3233614
P	-2.1392081	0.8683558	-1.4014276
C	3.1872911	-0.5736067	-2.8915000
C	2.3368848	-2.6987037	-1.2788958
H	1.8864822	-3.0517990	-0.3495060
N	3.7452763	-3.0605929	-1.3228635
H	1.8023084	-3.1666582	-2.1082693
H	3.3088459	0.5019349	-3.0326902
H	2.6762089	-0.9751725	-3.7691612
N	4.4868370	-1.2093082	-2.7323189
C	3.3438950	-0.3511710	-0.1023880
H	3.4763182	0.7324934	-0.1554827
N	4.6210062	-1.0198775	-0.3006167
H	2.9407313	-0.5936782	0.8839296
C	-2.3368848	2.6987037	-1.2788958
H	-1.8023084	3.1666582	-2.1082693
H	-1.8864822	3.0517990	-0.3495060
N	-3.7452763	3.0605929	-1.3228635
C	-3.3438950	0.3511710	-0.1023880
H	-3.4763182	-0.7324934	-0.1554827
N	-4.6210062	1.0198775	-0.3006167
H	-2.9407313	0.5936782	0.8839296
C	-3.1872911	0.5736067	-2.8915000
H	-3.3088459	-0.5019349	-3.0326902
H	-2.6762089	0.9751725	-3.7691612
N	-4.4868370	1.2093082	-2.7323189
C	4.3677495	-2.6552163	-2.5788519
H	5.3736049	-3.0805709	-2.6084563
H	3.7897927	-3.0595339	-3.4124355
C	-5.2167179	0.6750388	-1.5875546
H	-5.2778101	-0.4117470	-1.6745375
H	-6.2279731	1.0877705	-1.6116269
C	-4.3677495	2.6552163	-2.5788519
H	-5.3736049	3.0805709	-2.6084563
H	-3.7897927	3.0595339	-3.4124355
C	-4.4917257	2.4709950	-0.2171900
H	-5.4961979	2.9001257	-0.2208339
H	-4.0031612	2.7324907	0.7231180
C	5.2167179	-0.6750388	-1.5875546
H	5.2778101	0.4117470	-1.6745375
H	6.2279731	-1.0877705	-1.6116269
C	4.4917257	-2.4709950	-0.2171900
H	4.0031612	-2.7324907	0.7231180
H	5.4961979	-2.9001257	-0.2208339
Au	0.0000000	0.0000000	1.7697213
N	-0.3759669	1.9709538	1.7772843
N	0.3759669	-1.9709538	1.7772843
C	0.3511742	2.9657418	1.1977092
N	-0.1554440	4.1744626	1.3378668

molecules MDPI

C	-1.2987830	3.9619867	2.0582825
C	-1.4391251	2.6028776	2.3361539
C	-0.3511742	-2.9657418	1.1977092
C	1.4391251	-2.6028776	2.3361539
C	1.2987830	-3.9619867	2.0582825
N	0.1554440	-4.1744626	1.3378668
N	2.4514207	-2.0179722	3.0246006
C	3.3412411	-2.8652942	3.4371510
N	3.2935719	-4.1998239	3.2122775
C	2.2880714	-4.8804540	2.5184764
C	-2.2880714	4.8804540	2.5184764
N	-2.4514207	2.0179722	3.0246006
N	-3.2935719	4.1998239	3.2122775
C	-3.3412411	2.8652942	3.4371510
H	-4.0430933	4.7713189	3.5787323
H	4.0430933	-4.7713189	3.5787323
O	2.3426050	-6.0972264	2.3765679
O	-2.3426050	6.0972264	2.3765679
H	4.2010272	-2.5190096	3.9992067
H	-1.2701676	-2.7561624	0.6683713
H	-4.2010272	2.5190096	3.9992067
H	1.2701676	2.7561624	0.6683713
